# Spatial metabolomics reveals upregulation of several pyrophosphate-producing pathways in cortical bone of *Hyp* mice

**DOI:** 10.1172/jci.insight.162138

**Published:** 2022-10-24

**Authors:** Achim Buck, Verena M. Prade, Thomas Kunzke, Reinhold G. Erben, Axel Walch

**Affiliations:** 1 Research Unit Analytical Pathology, Helmholtz Zentrum München, German Research Center for Environmental Health, Neuherberg, Germany.; 2 Department of Biomedical Sciences, University of Veterinary Medicine, Vienna, Austria.

**Keywords:** Bone Biology, Bone disease, Mouse models

## Abstract

Patients with the renal phosphate–wasting disease X-linked hypophosphatemia (XLH) and *Hyp* mice, the murine homolog of XLH, are characterized by loss-of-function mutations in phosphate-regulating endopeptidase homolog X-linked (*PHEX*), leading to excessive secretion of the bone-derived phosphotropic hormone FGF23. The mineralization defect in patients with XLH and *Hyp* mice is caused by a combination of hypophosphatemia and local accumulation of mineralization-inhibiting molecules in bone. However, the mechanism by which PHEX deficiency regulates bone cell metabolism remains elusive. Here, we used spatial metabolomics by employing matrix-assisted laser desorption/ionization (MALDI) Fourier-transform ion cyclotron resonance mass spectrometry imaging (MSI) of undecalcified bone cryosections to characterize in situ metabolic changes in bones of *Hyp* mice in a holistic, unbiased manner. We found complex changes in *Hyp* bone metabolism, including perturbations in pentose phosphate, purine, pyrimidine, and phospholipid metabolism. Importantly, our study identified an upregulation of several biochemical pathways involved in intra- and extracellular production of the mineralization inhibitor pyrophosphate in the bone matrix of *Hyp* mice. Our data emphasize the utility of MSI–based spatial metabolomics in bone research and provide holistic in situ insights as to how Phex deficiency–induced changes in biochemical pathways in bone cells are linked to impaired bone mineralization.

## Graphical abstract

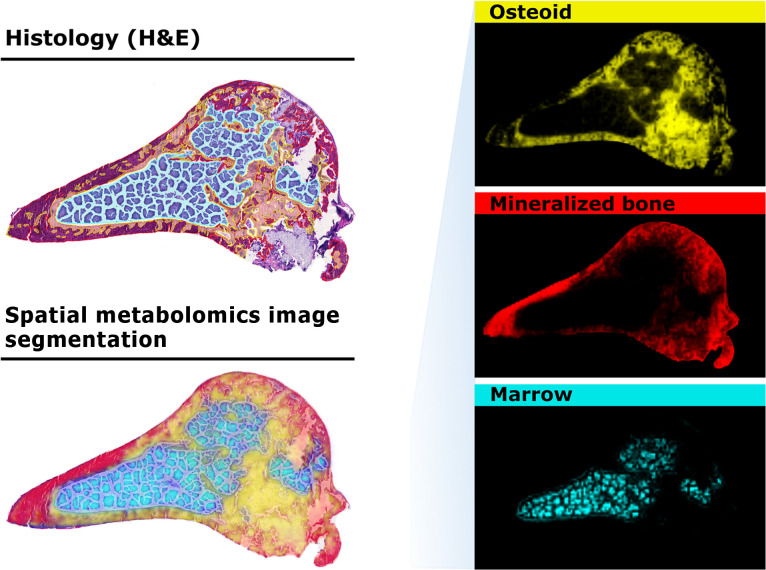

## Introduction

Many biological processes rely on tightly regulated phosphate levels, and deviation from the normal phosphate homeostasis leads to a variety of symptoms. Renal phosphate–wasting diseases, such as X-linked hypophosphatemia (XLH), result in chronic hypophosphatemia, which subsequently causes hypomineralization of bones, leading to rickets and osteomalacia (1). XLH is the most frequent form of inherited rickets in humans and is caused by inactivating mutations in the phosphate-regulating endopeptidase homolog X-linked (PHEX) gene (2–4). The most prominent clinical problems in patients with XLH, such as leg deformities, dental diseases, skeletal pain, and fractures, are caused by impaired bone mineralization (5). Patients with XLH exhibit elevated circulating intact levels of the bone-derived hormone FGF23, which is known to regulate phosphate homeostasis (5). Excessive FGF23 secretion is the main cause for renal phosphate wasting in patients with XLH (5). The murine homolog and animal model for XLH is *Hyp* (hypophosphatemia). Similar to patients with XLH, *Hyp* mice harbor a pathogenic loss-of-function variant of *Phex*, causing an XLH-like phenotype. *Hyp* mice are characterized by low serum phosphate and vitamin D hormone levels, increased serum levels of Fgf23, and impaired bone mineralization.

Why loss-of-function mutations in *PHEX* lead to excessive secretion of FGF23 and defective bone mineralization is only partially known. It is currently thought that impaired bone mineralization in patients with XLH and *Hyp* mice is characterized by at least 3 components: a) hypophosphatemia caused by FGF23-mediated renal phosphate wasting reduces the availability of phosphate for normal biomineralization; b) as shown in *Hyp* mice, increased osteocytic secretion of FGF23 leads to an auto/paracrine suppression of the pyrophosphate-degrading enzyme tissue nonspecific alkaline phosphatase (TNAP) and subsequent accumulation of the mineralization inhibitor pyrophosphate (PPi) (6); and c) PHEX deficiency is associated with the accumulation of osteopontin and so-called acidic serine aspartate-rich MEPE-associated motif (ASARM) peptides in the matrix, interfering with normal mineralization (7, 8). The relative contribution of these 3 processes to the impairment of bone mineralization in XLH is unknown. PPi is a small diphosphate molecule that is produced by osteoblasts during bone formation. The physiological mineralization of bone depends on a tight local balance between the extracellular levels of inorganic phosphate (Pi) and PPi (9). PPi is a potent inhibitor of mineralization, antagonizing the incorporation of Pi and calcium into nascent hydroxyapatite crystals, thereby preventing their growth and deposition onto collagen I (10). In addition, PPi upregulates osteopontin and inhibits TNAP activity (11). Hence, dysregulation of PPi metabolism directly affects bone mineralization. Whether PHEX deficiency alters PPi metabolism in bone cells in ways other than suppressing its degradation is currently unknown.

Our knowledge of the molecular mechanisms by which PHEX deficiency induces multifaceted metabolic alterations in bone cells is still fragmentary. Phex deficiency is associated with transcriptional changes in multiple genes in bones of *Hyp* mice (12). Moreover, osteoblasts isolated from *Hyp* mice are characterized by complex changes in gene expression (13) and cell metabolism (14–16). However, the in situ metabolism within the bone tissue in *Hyp* mice or patients with XLH remains unknown. This is an important gap in knowledge because a comprehensive characterization of biochemical pathways altered by Phex deficiency in bone cells within their natural environment is key to uncovering the links between biochemical changes and impaired bone mineralization in *Hyp* mice and patients with XLH.

In the current study, we asked whether high mass resolution matrix-assisted laser desorption/ionization (MALDI) Fourier-transform ion cyclotron resonance mass spectrometry imaging (MSI) could be employed to gain insights into the in situ metabolic alterations in *Hyp* bones and could be used to further explore the mechanisms underlying dysregulated PPi metabolism in *Phex*-deficient mice. MSI is used for in situ spatial metabolomics analysis to visualize the distribution of thousands of molecules within a tissue section. The technology is frequently used in soft tissues, but its application in hard tissues such as bones is scarce (17–20). MSI of bones has been achieved in methodological proof-of-principle experiments by following specifically developed protocols for bone tissue preprocessing and sectioning. However, there have not yet been any systematic knowledge-generating applications of MSI in bone disease models.

Here, we provide global insights into the in situ metabolic nature of mineralized and unmineralized bone tissue from *Hyp* mice and WT controls, employing MALDI imaging–based spatial metabolomics in cryosections of undecalcified bones. We found an upregulation of several PPi-producing biochemical pathways; an aberrant glycan metabolism; and perturbations in pentose phosphate, glycolysis/gluconeogenesis, cysteine and methionine, purine and pyrimidine, galactose, phospholipid, and inositol-related metabolism in cortical bone of *Hyp* mice. These findings provide a comprehensive understanding of biochemical changes in the bones of *Hyp* mice.

## Results

### Spatial multivariate analysis reveals metabolic differences between distinct bone tissue compartments.

**Figure 1:**
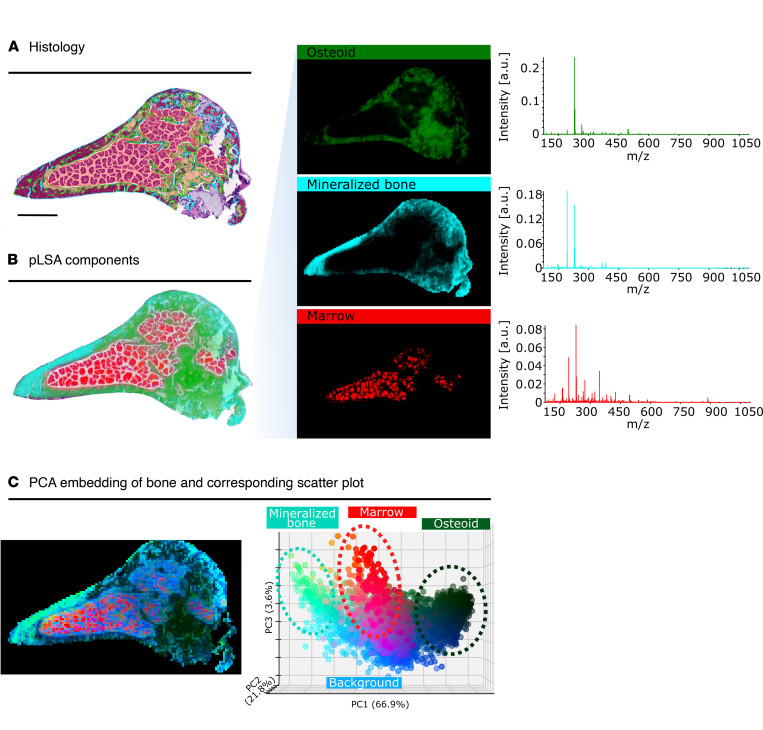
Histology of an undecalcified cryosection of a *Hyp* mouse femur and metabolic differences within the bone revealed by MALDI mass spectrometry imaging. (**A**) H&E staining of an undecalcified cryosection of a distal femur from a male 3-month-old *Hyp* mouse with annotated mineralized cortical bone (cyan), unmineralized bone matrix (osteoid, dark green), and bone marrow (red). Not annotated is cartilage. The scanned H&E image was annotated in QuPath (v. 0.1.2) (52). (**B**) Probabilistic latent semantic analysis (pLSA) components generated from spatial metabolomics show differences between the anatomical features and their corresponding characteristic mass spectra on the right. (**C**) Unsupervised principal component analysis (PCA) of the same section showing the PCA embedding as well as the corresponding scatter plot. Scale bar: 1 mm.

To assess the ability of MALDI imaging of undecalcified bone sections to resolve the metabolic differences of the individual bone-specific compartments, we initially performed a probabilistic latent semantic analysis (pLSA) and a principal component analysis (PCA) on a femur section from a *Hyp* mouse. pLSA is a supervised method for component analysis that requires prior knowledge of the number of tissue compartments (we assumed 3 — mineralized bone, osteoid, and bone marrow), but allows identification of a metabolic signature per compartment. PCA is an unsupervised dimensionality reduction method commonly used to visualize metabolic differences or similarities between any number of cell or tissue types, which is often used for subsequent tissue segmentation/clustering. Both algorithms allowed clear spatial metabolic distinction of tissue compartments, which could be histologically assigned to osteoid, mineralized cortical bone, and bone marrow (Figure 1).

Using pLSA, the spatial tissue compartments were evaluated in terms of their corresponding molecular signatures. Peak picking (S/N 3) yielded 565 metabolites associated with osteoid (dark green), 374 metabolites associated with mineralized cortical bone (cyan), and 702 metabolites associated with bone marrow (red) (Figure 1, A and B). Another component representing a background signature was not considered for further evaluation. The unsupervised PCA was performed on the same bone section and confirmed the metabolic differences of the anatomical features (Figure 1C). The PCA scatter plot reveals 3 metabolically distinct groups of pixels representing osteoid and cartilage (dark green), mineralized cortical bone (cyan), and bone marrow (red), which converge with a group of pixels representing the background signature (blue). For comparison, pLSA of a WT bone section was performed, distinguishing between mineralized cortical bone and bone marrow (Supplemental Figure 1; supplemental material available online with this article; https://doi.org/10.1172/jci.insight.162138DS1).

Collectively, these results clearly indicate that MALDI MSI of undecalcified bone cryosections is able to reliably pick up metabolic signatures of different cell and tissue types in bone.

### The cortical bone metabolic profile is altered in Hyp mice.

**Figure 2:**
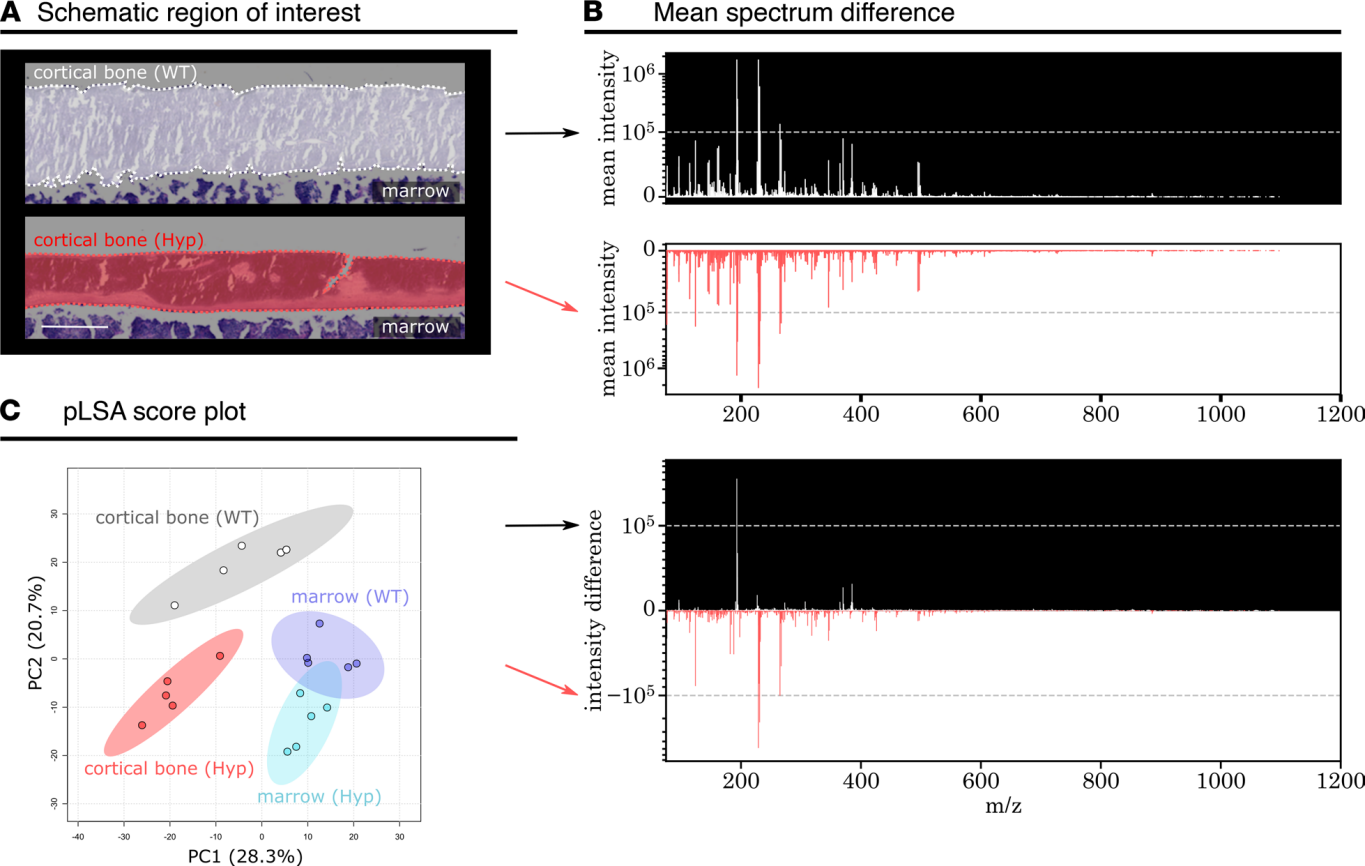
Metabolic difference of the cortical bone in WT mice and *Hyp* mice. (**A**) Schematic representation of the region of interest (ROI) analyzed in WT (white) and *Hyp* (red) femoral shafts. The ROI includes mineralized bone and osteoid. (**B**) Juxtaposition of the overall mean spectrum from WT and *Hyp* cortical bone ROIs selected in **A**, represented as mean intensity and intensity difference. (**C**) pLSA score plot revealing metabolic differences between cortical bone ROIs of WT and *Hyp* mice (*n* = 5 mice per group). Each data point in the pLSA score plot represents the mean spectrum of an annotated ROI from 1 mouse bone tissue section. Bone marrow was included as a control and is metabolically similar between WT and *Hyp* mice. Scale bar: 200 μm.

To assess overall metabolic differences in bones of *Hyp* and WT mice, cortical bone of the femoral shaft, including mineralized tissue and osteoid, was annotated and used for all subsequent analyses (Figure 2A). The calculated mean mass spectra of WT and *Hyp* bones indicated a distinctly different metabolic profile (Figure 2B). Subsequently, we used pLSA to visualize metabolic differences between bones of WT and *Hyp* mice (Figure 2C). The metabolic signatures of cortical bones were clearly distinct, whereas there was no clear metabolic difference between the bone marrow of WT and *Hyp* mice (Figure 2C).

### Metabolic pathway analysis shows major changes in cortical bone of Hyp mice.

**Figure 3:**
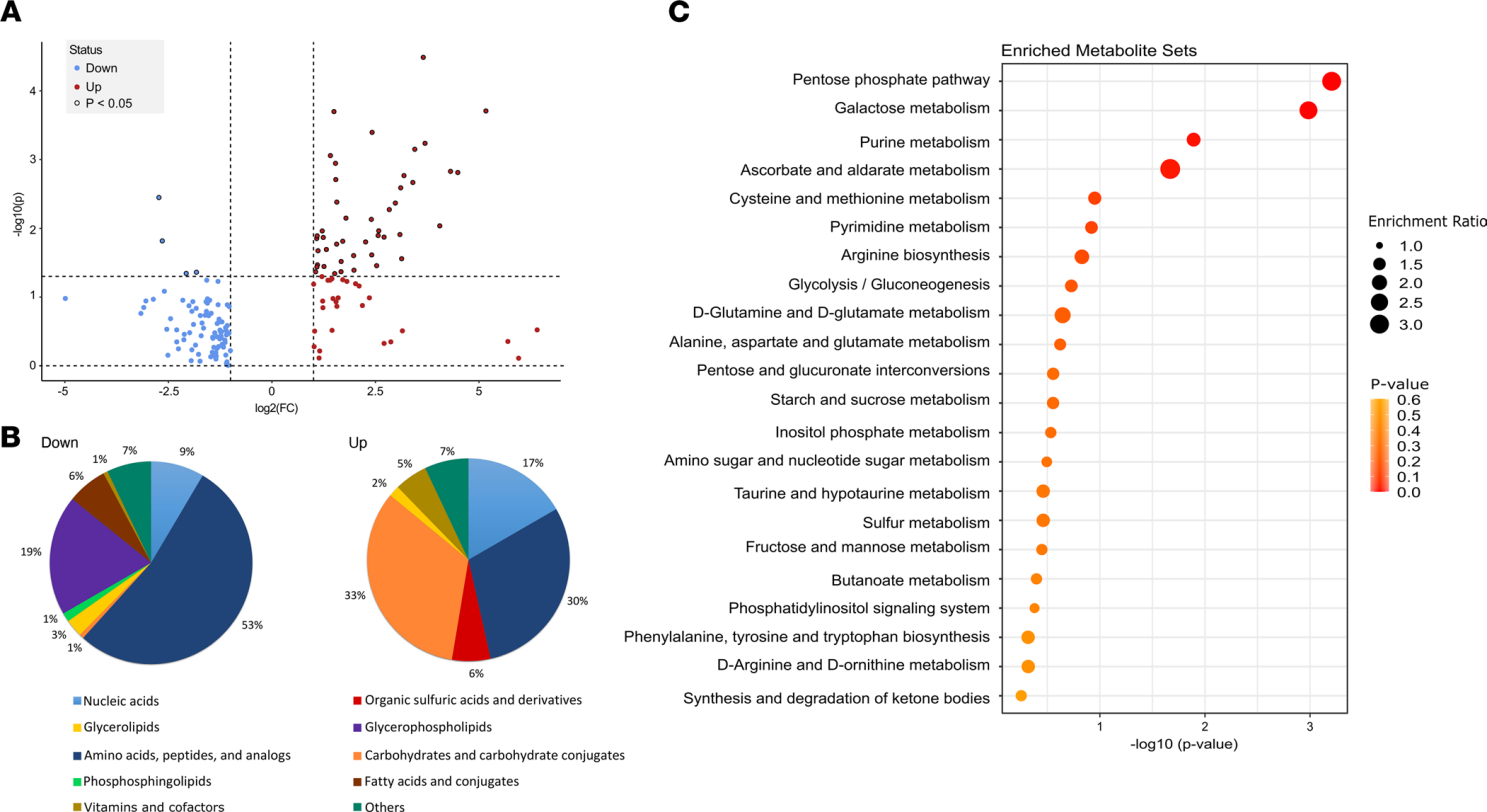
Pathway analysis revealing metabolic alterations between WT and *Hyp* cortical bone. (**A**) Volcano plot of the distribution of differentially abundant metabolites, mapping 160 at least log_2_ fold-changed metabolites with 87 metabolites of reduced (blue) and 73 metabolites of increased (red) abundance in *Hyp* cortical bone versus WT controls (log_2_ fold change cutoff value of ≥ 1 for upregulation or ≤ −1 for downregulation). (**B**) Pie charts of the percentages of log_2_ fold change metabolites (up/down) with metabolite class information from HMDB and KEGG databases. (**C**) Enrichment pathway analysis conducted by using functionally annotated log_2_ fold change metabolites. Dot plot showing the significantly changed pathways in *Hyp* cortical bone. The size of the circles per pathway set represents the enrichment ratio, and the color represents the *P* value.

To determine metabolites in cortical bone that were changed by inactivation of *Phex* in *Hyp* mice on a metabolome-wide basis, we performed pathway analysis. In comparison with WT, a total of 415 metabolites (log_2_ fold change, *P* < 0.05) showed different abundance in *Hyp* bones (Supplemental Table 1). Among these, 160 log_2_ fold-changed metabolites could be functionally annotated with 87 metabolites of reduced abundance and 73 metabolites of increased abundance in *Hyp* bones (Figure 3A and Supplemental Table 2). In the pie charts shown in Figure 3B, the differentially abundant metabolites were assigned to metabolic classes based on information from the Human Metabolome Database (HMDB) and Kyoto Encyclopedia of Genes and Genomes (KEGG) chemical taxonomy. *Hyp* cortical bones showed decreased levels of glycerophospholipids, phosphosphingolipids, fatty acids, amino acids, and peptides. Conversely, carbohydrates, nucleic acids, vitamins and cofactors, and organic sulfuric acids were increased in *Hyp* bones. Ranking of metabolites according to their impact on metabolic pathways identified major enrichments in the pentose phosphate pathway (PPP), galactose metabolism, purine metabolism, ascorbate and aldarate metabolism, cysteine and methionine metabolism, pyrimidine metabolism, and arginine metabolism, as well as in glycolysis and gluconeogenesis (Figure 3C).

**Figure 4:**
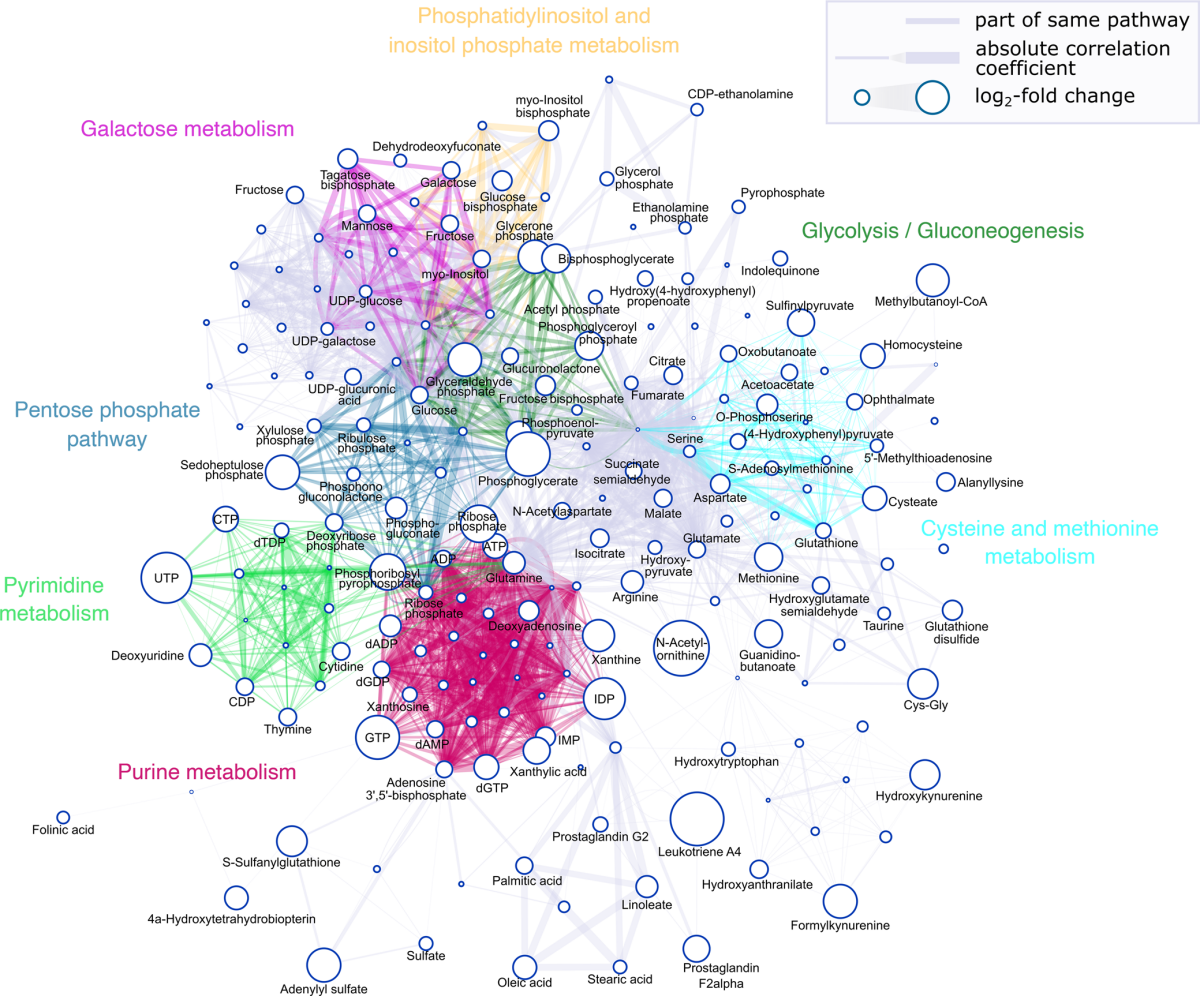
Spatial metabolomics pathway network. Nodes represent metabolites, with size reflecting the log_2_ fold change between WT and *Hyp* cortical bone. Node sizes increase with the log_2_ fold change, while the criteria for labeling nodes is a log_2_ fold change cutoff value of 1 or greater for upregulation or –1 or less for downregulation, respectively. Edges represent shared KEGG pathways, with the width representing the spatial correlation coefficient within compacta. Edge opacity increases with the correlation coefficient. Selected pathways are colored as follows: purine metabolism (red), pyrimidine metabolism (light green), pentose phosphate pathway (blue), galactose metabolism (magenta), glycolysis/gluconeogenesis (dark green), phosphatidylinositol and inositol phosphate metabolism (yellow), and cysteine and methionine metabolism (cyan).

One of the strengths of MSI is the possibility to perform a global metabolic biochemical network analysis, correlating the pattern of spatially colocalized metabolites. Figure 4 shows the spatial pathway network analysis in cortical bone of *Hyp* versus WT mice. Several perturbed pathways from the enrichment pathway analysis are highlighted in the spatial correlation network analysis by color coding (Figure 4). It is evident that metabolites from altered pathways in *Hyp* bones showed distinct spatial colocalization. The PPP was found to be upregulated in the cortical bone of *Hyp* mice compared with WT. In the PPP network, the following metabolites showed greater than log_2_ fold change (values are displayed in parentheses): glyceraldehyde phosphate (3.18), phosphoglycerate (4.28), deoxyribose phosphate (1.53), ribose phosphate (3.57), phosphogluconate (1.94), xylulose phosphate (1.16), ribulose phosphate (1.16), phosphonogluconolactone (1.10), sedoheptulose phosphate (3.25), and phosphoribosyl pyrophosphate (3.46). The pentose phosphate metabolite phosphoribosyl pyrophosphate is important for the formation of purine and pyrimidine nucleotides. Both purine metabolism and pyrimidine metabolism were found to be upregulated in *Hyp* bone. In the network, the ribonucleotide triphosphate pool of ATP (2.29), CTP (2.34), GTP (4.23), and UTP (4.95) was increased, whereas the size of the deoxyribonucleotide pool showed varying changes in dAMP (–1.47), dADP (–1.94), dGDP (1.44), dGTP (2.29), and dTDP (–1.23). In glycolysis and gluconeogenesis, glucose (1.49) levels were higher in *Hyp* bone compared with WT. Moreover, several glycolytic intermediates, such as fructose bisphosphate (1.77), glycerone phosphate (3.18), glyceraldehyde phosphate (3.18), bisphosphoglycerate (2.69), phosphoglycerate (4.28), and phosphoenolpyruvate (2.38), were increased in abundance. Beyond the core glycolysis pathway, several glycolytic intermediates can be metabolized by means of alternative mechanisms, including shunting glucose 6-phosphate through the PPP that is critical for synthesis of nucleotides and lipids or by the conversion of fructose-6-phosphate via the hexosamine biosynthetic pathway. For instance, the hexosamine biosynthetic pathway provides uridine diphosphate glucose–N-acetyl-glucosamine (UDP-GlcNAc) (0.34) units for the synthesis of several glycoconjugates. Other UDP sugars needed for the synthesis of glycosaminoglycans, such as UDP-glucose (1.01) and UDP-glucuronic acid (1.41), were increased in *Hyp* bone. Additional pathways altered between *Hyp* and WT included the galactose metabolism and the inositol-related pathways, with changes in galactose (1.49), glycerone phosphate (3.18), myo-inositol (1.49), and myo-inositol bisphosphate (1.77). Furthermore, the network analysis showed alterations in methionine and cysteine metabolism, with changed metabolite levels for methionine (2.62), cysteate (2.23), homocysteine (–2.25), S-adenosylmethionine (1.19), O-phosphoserine (1.89), aspartate (1.73), oxobutanoate (–1.41), sulfinyl pyruvate (–2.51), 5′-methylthioadenosine (–1.088), and glutathione (1.32).

### Glycan fragment signature is altered in cortical bone of Hyp mice.

**Figure 5:**
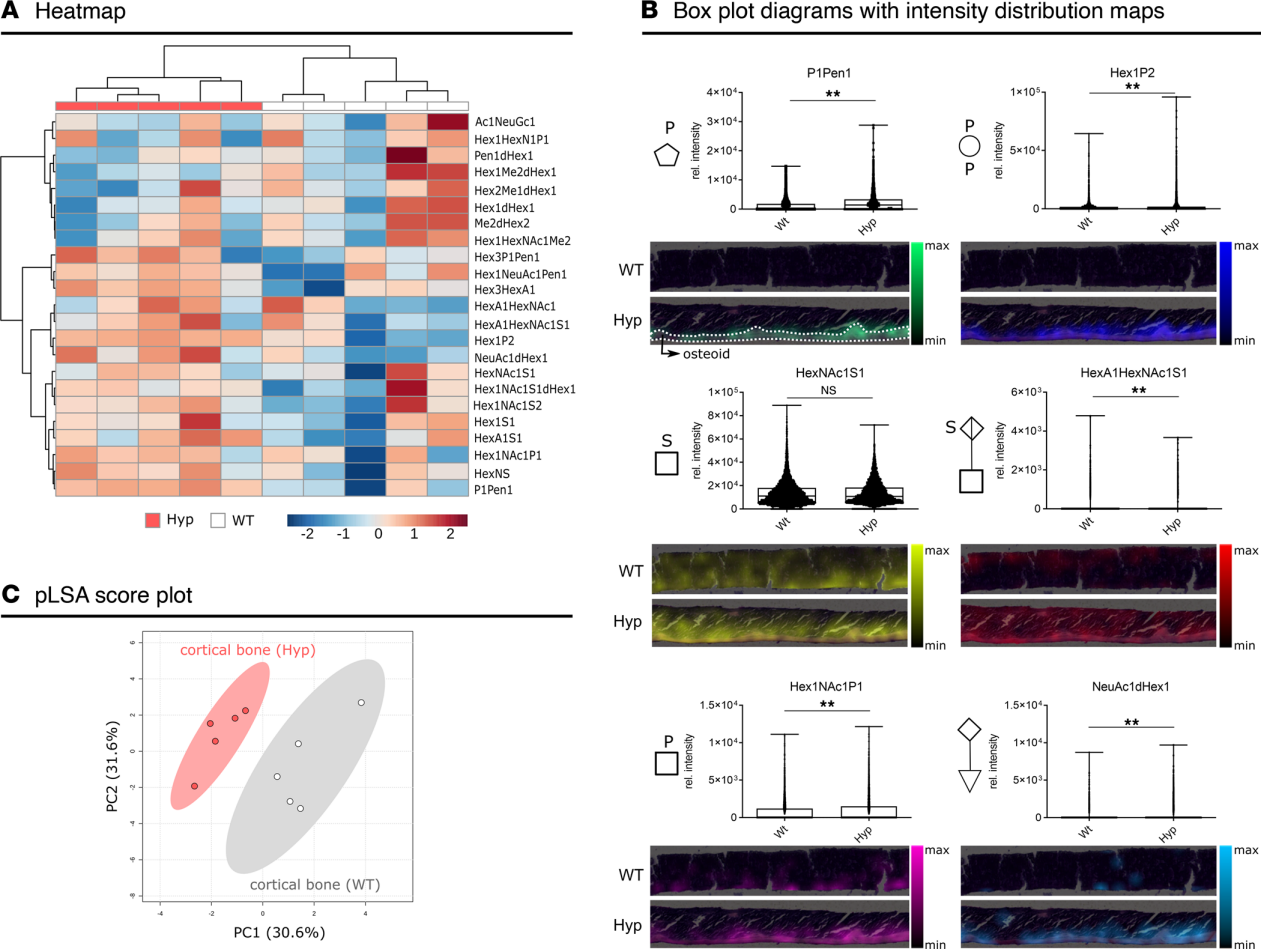
Aberrant glycan metabolism in the bone matrix of *Hyp* mice. (**A**) Heatmap of annotated glycan fragments in the mass range of *m/z* 75–1100 in cortical bone demonstrates different glycan expression patterns between *Hyp* and WT mice. (**B**) Bar graphs and intensity distribution maps of distinct glycan fragments assigned to individual colors in WT and *Hyp* bone sections. Annotation was performed in GlycoWorkbench (version 2.1, build 146). Box plots display the median and whiskers range from minimum to maximum for pixel-wise intensity distributions (*n* = 5 mice per group; *P* < 0.01 by Mann-Whitney *U* test). (**C**) pLSA score plot allows the separation of *Hyp* and WT cortical bone on the basis of the detected glycan fragments (*n* = 5 mice per group). dHex, deoxyhexose; Hex, hexose; HexA, hexuronic acid; HexAc, hexose acetate; HexAS, sulfated hexuronic acid; HexN, hexosamine; HexNAc, N-acetylhexosamine; HexNAcS, N-acetylhexosamine sulfate; HexP, hexose phosphate; HexS, hexose sulfate; NeuAc, N-acetylneuraminic acid; PPen, pentose phosphate; PenS, pentose sulfate.

In the spatial metabolomics pathway analysis (Figure 4), we found major changes in glycosaminoglycan metabolism. The abundances of UDP-glucuronic acid and UDP-GlcNAc, both components of glycosaminoglycans and proteoglycans in the extracellular matrix, were significantly higher in *Hyp* bone compared with WT (Supplemental Figure 2). Because glycans are not well represented in metabolic databases, but are important constituents of bone matrix, we mined our data set for detectable natively occurring glycans and glycan fragments using GlycoWorkbench. A total of 19 glycan fragments were found to be significantly different, whereas 4 glycans, HexA1S1, HexNAc1S1, Ac1NeuGc, and Hex1NAc1S1dHex1, showed no significant changes in cortical bone of *Hyp* versus WT mice (Figure 5A). Glycan fragments with different molecular modifications, such as acylation, phosphorylation, and sulfation, were associated with altered distribution patterns in WT and *Hyp* bones (Figure 5B). Phosphorylated fragments, such as pentose phosphate (P1Pen1), hexose bisphosphate (Hex1P2), and N-acetylhexosamine phosphate (Hex1NAcP1), were of increased abundance in the osteoid of *Hyp* bones. In addition, the sialylated N-glycan NeuAc1dHex1 was increased in the osteoid of *Hyp* bones (Figure 5B). In contrast, the sulfated glycosaminoglycan component N-acetylhexosamine sulfate (HexNAcS) was not different between groups. Interestingly, the glycosaminoglycan HexA1HexNAc1S1, the repeating disaccharide in chondroitin sulfate, which is a major component of the extracellular matrix (21), was significantly increased in *Hyp* as compared with WT cortical bone. A partial pLSA allowed the separation of *Hyp* and WT cortical bone on the basis of the detected glycan fragments (Figure 5C). The highlighted differences in N-linked glycan metabolism were particularly evident in the osteoid of *Hyp* mice, with hyperglycosylation of phosphate-modified glycans compared with WT controls. In summary, our data suggest that an aberrant glycan metabolism is a universal feature in the bone matrix of *Hyp* mice.

### Extracellular and intracellular PPi production pathways are upregulated in Hyp bones.

**Figure 6:**
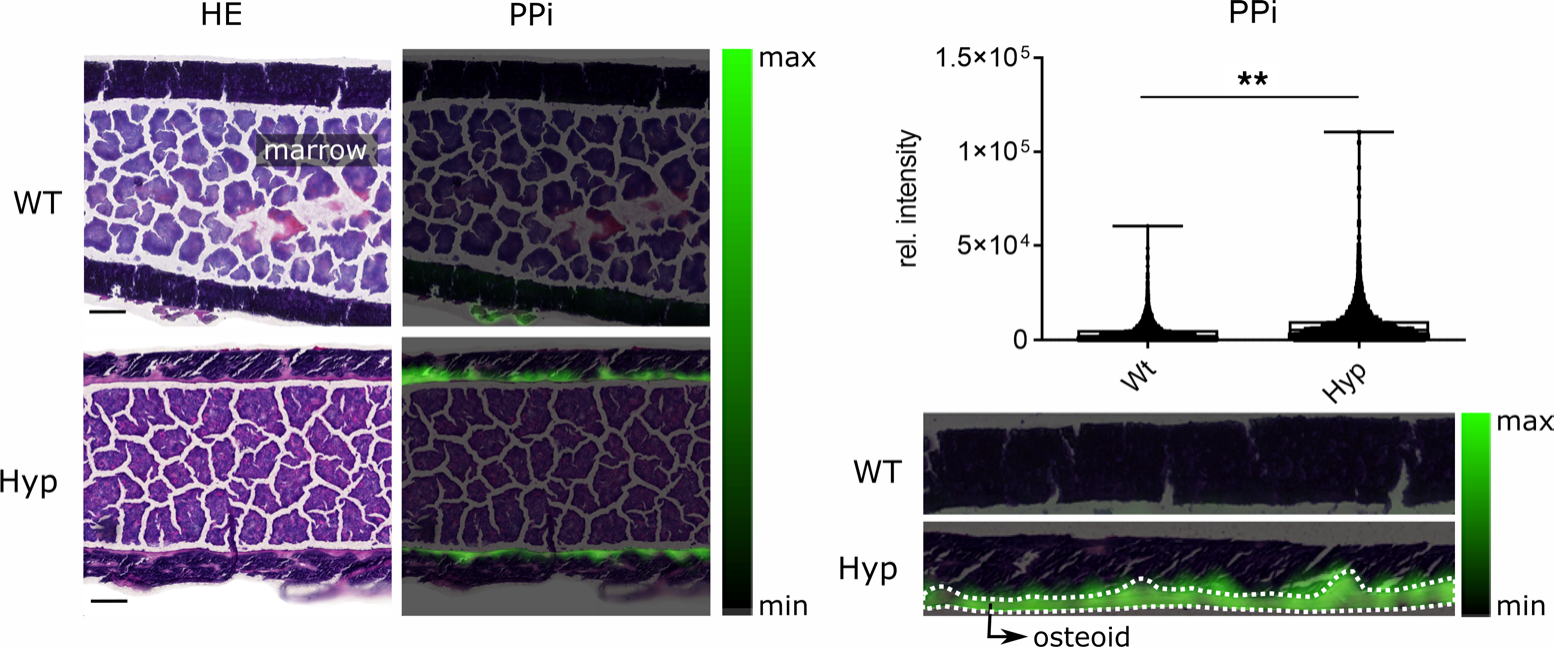
Increased abundance of pyrophosphate in bones of *Hyp* mice. The pyrophosphate (PPi) signal colored in green is of significantly higher abundance in the bone matrix of *Hyp* compared with WT mice. For better visualization, the PPi signal (left side) of the bone marrow in femoral shafts was excluded. Higher magnification images (right side) show that the PPi signals are specifically located in the osteoid layer of *Hyp* bones. Box plots display the median, and whiskers range from minimum to maximum for pixel-wise intensity distributions (*n* = 5 mice per group; *P* < 0.01 by Mann-Whitney *U* test). Scale bar: 200 μm. The same representative WT and *Hyp* sections are shown in Figure 5B and Figure 7.

In earlier biochemical analyses, we have shown increased PPi concentrations in extracts from femora of *Hyp* mice relative to WT controls (6). However, the distribution and localization of PPi in *Hyp* bones has been unknown so far. PPi is linked in the KEGG database to the pathway of oxidative phosphorylation, which was not particularly highlighted in the spatial correlation network (Figure 4). However, when we focused on the spatial localization of PPi in the MSI data set, we found the metabolite highly enriched in the bone matrix of *Hyp* mice (Figure 6). In contrast, PPi could not or only poorly be detected in the calcified regions of *Hyp* or WT bones (Figure 6).

**Figure 7:**
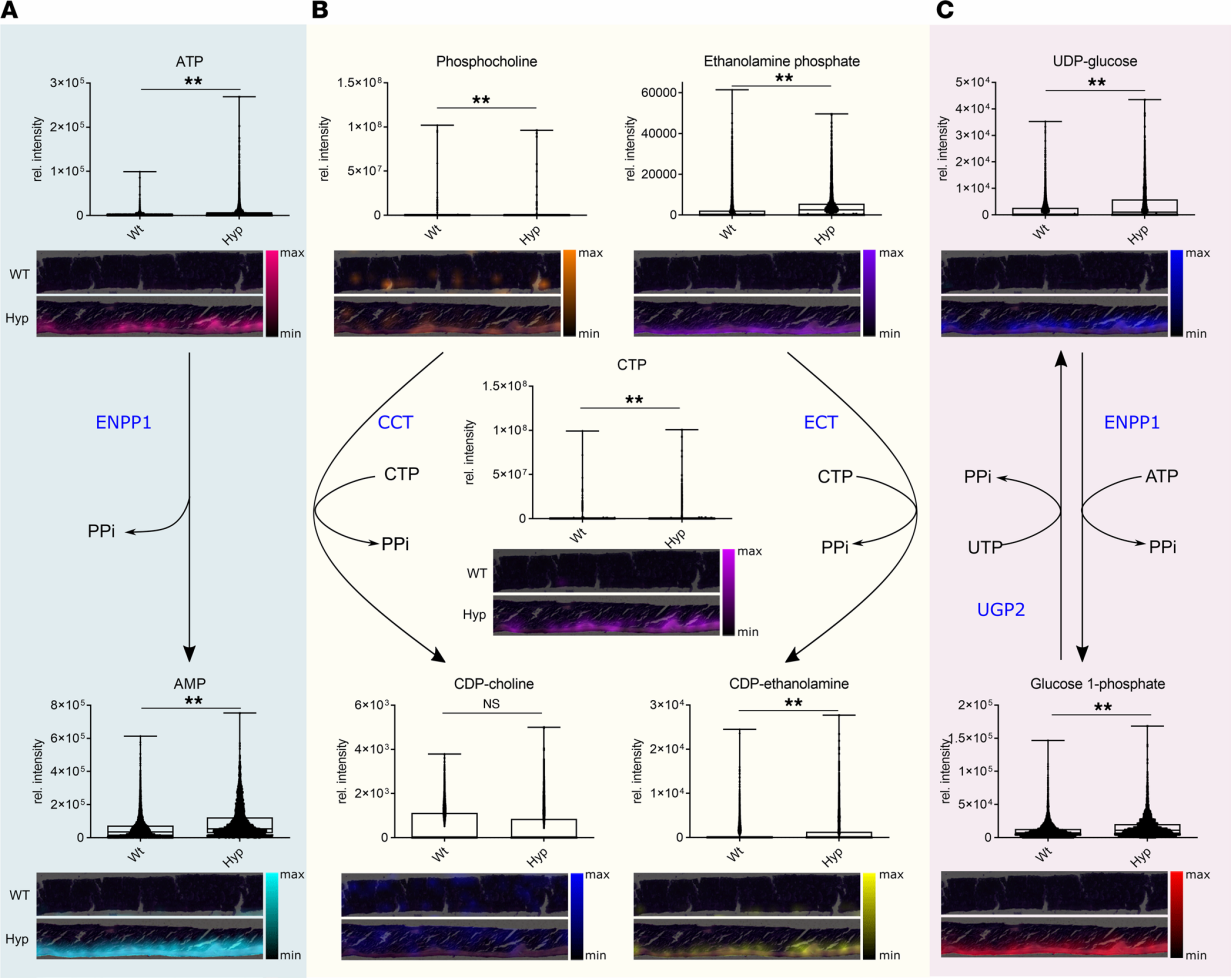
Pathways involved in PPi synthesis based on KEGG metabolic pathway maps in WT and *Hyp* bones. Representative visualization and intensity distribution maps of distinct metabolites assigned to individual colors in WT and *Hyp* bone sections. (**A**) The enzyme ENPP1 involved in HA crystallization in the extracellular space is responsible for the synthesis of PPi from ATP. Its substrate ATP and the product AMP were elevated in *Hyp* bones. (**B**) The CDP-choline pathway of the Kennedy pathway is presented on the left-hand side, while the other half of the Kennedy pathway on the right represents the CDP-ethanolamine pathway. PPi is released as a byproduct of enzymatic reactions in both branches of the Kennedy pathway, which is responsible for the de novo synthesis of phosphatidylcholine. (**C**) Metabolic steps in glycogenesis and glycogenolysis releasing PPi. In glycogen synthesis (glycogenesis) UDP-glucose pyrophosphorylase 2 (UGP2) catalyzes the transformation of G1P and UTP to UDP-glucose and PPi. In glycogen degradation (glycogenolysis), ENPP1 catalyzes the dephosphorylation of UDP-glucose into glucose 1-phosphate and PPi. Box plots display the median, and whiskers range from minimum to maximum for pixel-wise intensity distributions (*n* = 5 mice per group; *P* < 0.01 by Mann-Whitney *U* test). Scale bar: 200 μm. CDP, cytidine diphosphate; CTP, cytidine triphosphate; E/CCT, ethanolamine/choline phosphate cytidylyltransferase; ENPP1, ectonucleotide pyrophosphatase/phosphodiesterase 1; PPi, pyrophosphate; UDP, uridine diphosphate; UGP2, UDP-glucose pyrophosphorylase 2; UTP, uridine triphosphate. The same representative WT and *Hyp* sections are shown in Figure 5B and Figure 6.

Next, we examined the biochemical pathways regulating PPi abundance. The levels of PPi can be regulated extracellularly and intracellularly by different enzymatic processes. Extracellularly, PPi is produced by direct cleavage of the phosphodiester bond of ATP by the plasma membrane protein ectonucleotide pyrophosphatase/phosphodiesterase 1 (ENPP1) (22). Mitochondria-derived ATP is the major substrate for the nucleotide triphosphate-mediated generation of PPi. The amounts of the ATP substrate and the AMP product were significantly increased in the osteoid of *Hyp* relative to WT bones (Figure 7A). In contrast, the calcified compartment of the matrix showed low levels of the nucleotides in both genotypes (Figure 7A). These data suggest that ATP-mediated extracellular production of PPi is upregulated in the osteoid of *Hyp* mice.

To what extent intracellular pathways, in which PPi is formed as a byproduct of enzymatic reactions, may contribute to elevated PPi production in *Hyp* bones is unknown. In the latter case, it was previously thought that the intra- to extracellular channeling of PPi is mediated by the transmembrane protein progressive ankylosis protein homolog (ANKH) (23). However, it was shown recently that the main function of ANKH is the transport of ATP, not that of PPi (24). Therefore, it is currently unclear how intracellularly produced PPi is transported to the extracellular space. Intracellular routes of PPi production include the 2 parallel branches of the Kennedy pathway to synthesize CDP-choline and CDP-ethanolamine (Figure 7B) (25). In the CDP-choline pathway, the choline phosphate cytidylyltransferase (CCT) uses CTP to convert phosphocholine into CDP-choline, releasing PPi. Similarly, PPi is released via the distinct CDP-ethanolamine pathway. CDP-ethanolamine is synthesized by a reaction of ethanolamine phosphate together with CTP by ethanolamine phosphate cytidylyltransferase (ECT). In addition, in glycogen degradation, ENPP1 catalyzes the dephosphorylation of UDP-glucose into glucose 1-phosphate and PPi (Figure 7C) (26, 27). Vice versa, UDP-glucose pyrophosphorylase 2 (UGP2) catalyzes the transformation of glucose 1-phosphate and UTP to UDP-glucose and PPi during glycogen synthesis. With the exception of CDP-choline, the abundance of all metabolites associated with intracellular production of PPi was distinctly upregulated in the osteoid layer of *Hyp* relative to WT femora (Figure 7, B and C), suggesting that not only extracellular but also intracellular PPi production was higher in *Hyp* versus WT bones.

## Discussion

In this study, we provided a global, unbiased in situ analysis of metabolic changes in bones of *Hyp* mice, employing MALDI imaging of undecalcified bone cryosections. We found complex metabolic alterations in *Hyp* bones, such as perturbations in pentose phosphate, glycolysis/gluconeogenesis, cysteine and methionine, purine and pyrimidine, galactose, phospholipid, and inositol-related metabolism. In addition, our study identified an upregulation of several biochemical pathways involved in intra- and extracellular production of the mineralization inhibitor PPi as well as an aberrant glycan metabolism in the bone matrix of *Hyp* mice. Our data emphasize the applicability of MALDI imaging to answer biological questions in bone research.

Earlier biochemical studies have shown a dysregulated PPP and a markedly higher rate of gluconeogenesis in osteoblasts of *Hyp* mice (28–30). Our in situ spatial metabolomics approach corroborated these earlier results from cell culture experiments. The PPP is a fundamental component of cellular metabolism and is important for the maintenance of carbon homeostasis; for nucleotide, lipid, and amino acid biosynthesis; for the provision of reducing molecules in cell metabolism; and for counteracting oxidative stress. Of interest, the upregulation in PPP was associated with an altered ribonucleotide triphosphate pool in *Hyp* bones, whereas the abundance of phospholipids was decreased. In addition, our data revealed previously unknown changes in several other biochemical pathways in bones of *Hyp* mice, in particular galactose, cysteine and methionine, and inositol-related metabolism, adding a global perspective to what is known about metabolic changes in bone cells and extracellular bone matrix of *Hyp* mice.

An interesting finding in our study was the aberrant glycan metabolism in bones of *Hyp* mice. We found hyperglycosylation of phosphate-modified N-linked glycans and a higher abundance of the sialylated N-glycan NeuAc1dHex1 and of the glycosaminoglycan HexA1HexNAc1S1 (repeating disaccharide structure in chondroitin sulfate) in the osteoid of *Hyp* bones. N-linked glycan biosynthesis is connected with glucose metabolism by means of the shared glycolytic substrate fructose-6-phosphate (14). Fructose-6-phosphate is required for the synthesis of UDP-GlcNAc through the hexosamine pathway (15). The biosynthesis of the extracellular matrix is a dynamic process strictly influenced by cell metabolism and the availability of energy substrates, and it requires adequate levels of the precursors UDP-GlcNAc and UDP-glucuronic acid. Notably, the UDP sugars were found to be more abundant in *Hyp* bones compared with WT bones. Chondroitin sulfate is a strongly charged polyanion composed of a long unbranched polysaccharide chain with a repeating disaccharide structure of sulfated N-acetylgalactosamine and glucuronic acid. The major proteoglycans in the mineralized bone matrix are biglycan and decorin, which contain high levels of covalently bound chondroitin sulfate chains (16). In agreement with our data, an upregulation of biglycan was demonstrated previously in *Hyp* mice (31). The higher abundance of chondroitin sulfate in the *Hyp* bone matrix may contribute to the mineralization defect, because it has been shown that high concentrations of biglycan can inhibit the growth and proliferation of mineral crystals in vitro in gelatin gels (32). Although the molecular mechanisms are still incompletely understood, our study demonstrated that Phex deficiency induced complex changes in the glycan signature of the bone matrix in situ.

Impaired bone mineralization is a key feature in *Hyp* mice and patients with XLH. As mentioned above, it is currently thought that the mineralization defect is caused by a combination of hypophosphatemia and increased abundance of mineralization-inhibiting molecules, such as osteopontin, ASARM peptides, and PPi in the bone matrix. Hypophosphatemia in *Hyp* mice and patients with XLH is caused by elevated circulating intact FGF23, leading to renal phosphate wasting and suppression of vitamin D hormone production. The recent advent of anti-FGF23 antibody therapy in patients with XLH has enabled the effective treatment of hypophosphatemia, and anti-FGF23 therapy significantly improves the bone phenotype in patients with XLH (33, 34) and in *Hyp* mice (35). However, it remains unclear to what extent correction of hypophosphatemia is able to rescue the mineralization defect because the effect of anti-FGF23 therapy on the local levels of mineralization inhibitors, such as ASARM peptides, osteopontin, and PPi, is not known. Bone mineralization is a complex process that is regulated by the balance between mineralization inhibitors and the enzymes that degrade them (36, 37). It involves small molecule mineralization inhibitors, such as PPi; noncollagenous extracellular matrix proteins, such as osteopontin; and their derived bioactive peptides, such as ASARM peptides; and matrix vesicles (36, 37). The matrix vesicles are released by osteoblasts. TNAP, an enzyme essential for bone mineralization, is enriched in the membrane of these vesicles (38–40). The main role of TNAP for bone mineralization is the hydrolysis of the potent mineralization inhibitor PPi to Pi (41, 42). After cleavage of PPi, the precipitation of hydroxyapatite crystals depends on adequate levels of calcium and inorganic phosphate in the matrix vesicles and the extracellular matrix (43).

We have previously shown that increased levels of PPi in bone contribute to the mineralization defect in *Hyp* mice (6). In this context, we reported that increased osteocytic secretion of FGF23 leads to an auto/paracrine suppression of *Tnap* transcription via an αKlotho-independent signaling pathway, with subsequent accumulation of PPi in *Hyp* bone (6). In the current study, spatial metabolomics uncovered that not only the degradation but also the production of PPi was altered in *Hyp* bones, leading to substantially elevated levels of PPi in the osteoid. We found an upregulation of not only ENPP1-mediated extracellular pathways of PPi production but also intracellular pathways releasing PPi, such as the Kennedy pathway (25, 44, 45), and ENPP1-mediated dephosphorylation of UDP-glucose in glycogen degradation (26, 27). The CDP-choline pathway, first identified by Eugene Kennedy in 1956, is the predominant mechanism by which mammalian cells synthesize phosphatidylcholine for incorporation into membranes or lipid-derived signaling molecules (25). The CDP-choline pathway represents one half of what is known as the Kennedy pathway. The other half is the CDP-ethanolamine pathway, which is responsible for the biosynthesis of the phospholipid product phosphatidylethanolamine. Ethanolamine phosphate is activated through a condensation reaction with CTP to form CDP-ethanolamine. The reaction is mediated by the cytosolic enzyme ECT. ECT is the rate-limiting enzyme in the CDP-ethanolamine pathway, which depends on the availability of both CDP-ethanolamine and diacylglycerol. Reciprocally, ethanolamine phosphate and phosphocholine can be hydrolyzed by the intracellular phosphoethanolamine/phosphocholine phosphatase (Phospho1), liberating Pi for the crystallization of hydroxyapatite. Both halves of the Kennedy pathway result in the intracellular release of PPi, and metabolites associated with the Kennedy pathway were distinctly upregulated in the osteoid layer of *Hyp* relative to WT bones. It is currently unknown whether the Kennedy pathway is also operative in matrix vesicles.

In conclusion, we herewith present the first study, to our knowledge, employing MALDI imaging–based spatial metabolomics to address a biological question in bone research. We found complex metabolic alterations in *Hyp* bones and identified an upregulation of several biochemical pathways involved in intra- and extracellular PPi production as well as an aberrant glycan metabolism in the bone matrix of *Hyp* mice. Our data may contribute to a more complete understanding of the pathways linking inactivating *Phex* mutations with intrinsic bone abnormalities and may provide a rich resource for the research community.

## Methods

### Animals.

Male WT controls and *Hyp* mice were bred by mating WT females with *Hyp* males on C57BL/6N background. Tail length at the time of weaning was used for genotyping. Animals were kept at 24°C with a 12-hour light/12-hour dark cycle and were fed a normal mouse diet (V1124-000, Sniff) containing 1.0% calcium, 0.7% phosphorus, and 1000 IU vitamin D/kg. At necropsy, femora from 3-month-old male WT and *Hyp* mice (*n* = 5 animals per group) were harvested under general anesthesia with ketamine/medetomidine (100/0.25 mg/kg, i.p.), and carefully cleaned from surrounding tissues. Immediately thereafter, the bones were put into embedding molds, covered with OCT compound (Sakura Finetek), and snap-frozen in liquid nitrogen.

### Spatial metabolomics.

First, 5 μm thick cryosections of undecalcified bones were prepared using a Leica CM 1950 cryostat with the help of a cryotape (Section Lab) as described (46). After sectioning, the tapes with the bone tissues were fixed to glass slides coated with indium-tin-oxide (Bruker Daltonik). The air-dried tissue sections were spray-coated with 10 mg/mL of 9-aminoacridine hydrochloride monohydrate matrix (Sigma-Aldrich) in 70% methanol using the SunCollect sprayer (Sunchrom). The matrix was applied in 8 passes (ascending flow rates of 10 μL/min, 20 μL/min, and 30 μL/min for layers 1–3; 40 μL/min for layers 4–8), using a line distance of 2 mm and a spray velocity of 900 mm/min.

Metabolites were detected in negative-ion mode on a 7T Solarix XR Fourier-transform ion cyclotron resonance mass spectrometer (Bruker Daltonik) equipped with a dual electrospray ionization–MALDI (ESI-MALDI) source and a SmartBeam-II Nd:YAG (355 nm) laser. Data acquisition parameters were specified in ftmsControl software 2.2 and flexImaging (v. 5.0) (Bruker Daltonik). Mass spectra were acquired covering *m/z* 75–1100. The instrument was calibrated externally with L-arginine in the ESI mode and internally using the 9-AA matrix ion signal (*m/z* 193.0771) as lock mass. The laser operated at a frequency of 1000 Hz using 100 laser shots per pixel with a pixel resolution of 50 μm.

### H&E staining and slide digitization.

After MALDI MSI, the 9-AA matrix was removed from the tissue section surface using 70% ethanol (Carl Roth) for 4 minutes, followed by H&E staining of the very same tissue sections. The H&E-stained tissue sections were cover-slipped and scanned with an AxioScan.Z1 digital slide scanner (Carl Zeiss) equipped with a 20× magnification objective. The visualization and export of the images to TIFF was done with the software ZEN 2.3 blue edition (Carl Zeiss).

### Peak picking and metabolite annotation.

MALDI MSI data were loaded into SCiLS Lab (v. 2020b Pro), and the digital stains were coregistered to the metabolomics data. The mass spectra were root-mean-square normalized, and 10,429 picked peaks were exported as imzML files for further data processing and subsequent bioinformatics data analysis with an in-house Python 3 pipeline as described previously (47). Osteoid and mineralized cortical bone regions were annotated. Metabolites were functionally annotated based on accurate mass matching of 4 ppm or less of the spectrometric *m/z* values to KEGG (48) and HMDB (49), allowing M-H, M-H_2_O, M+K-2H, M+Na-2H, and M+Cl as negative adducts. We excluded all irrelevant annotations (exogenous metabolites such as drugs and pesticides). For glycan annotation, GlycoWorkbench (version 2.1, build 146) was used (50).

### Spatial correlation metabolic networks.

Spatial correlation networks were created with Cytoscape (v. 3.7.2) (51). Nodes represent metabolites with a node size and color corresponding to the intensity log_2_ fold change between WT and *Hyp* cortical bone. Edges represent spatial correlations, with line opacity increasing with the correlation coefficient. Spatial correlations were calculated using Spearman’s rank correlation (Python 3.8.5, scipy v.1.6.0). Annotated metabolites with at least one significant correlation (*P* ≤ 0.05) are shown. The network was visualized using the Compound Spring Embedder layout using the absolute value of the correlation coefficient.

### Data availability.

The raw data that support the findings of this study will be made fully available upon acceptance of the paper (https://zenodo.org/record/7150208).

### Statistics.

Metabolites with significantly different intensity distributions between WT and *Hyp* cortical bone were identified with the 2-sided Mann-Whitney *U* test (Python 3.8.5, scipy v.1.6.0). Metabolites with significantly different intensity distributions between more than 2 WT and *Hyp* bone compartments were identified with the Kruskal-Wallis H-test (Python 3.8.5, scipy v.1.6.0), followed by the post hoc pairwise test for multiple comparisons of mean rank sums (Dunn’s test, scikit_posthoc v.0.6.6) while adjusting the *P* values with the Benjamini-Hochberg correction. *P* values equal or less than 0.05 were considered statistically significant. Multivariate analyses with unsupervised PCA and supervised pLSA were performed using the picked peaks as input to spatially characterize molecular features in bone and to determine differences between WT and *Hyp* groups. Additionally, individual bone sections were annotated and segmented into the 3 regions of osteoid, mineralized bone, and bone marrow.

The pLSA component analysis of the WT and *Hyp* bone was performed in SCiLS Lab. An identified background component was excluded from further analysis. The PCA was calculated using the Python package Sklearn (scikit-learn v.0.23.2). Heatmap-based clustering, volcano plot, and enrichment analyses were created using MetaboAnalyst 5.0 (http://www.metaboanalyst.ca).

### Study approval.

All animal procedures were undertaken in accordance with prevailing EU and national guidelines for animal care and welfare and approved by the Ethics and Animal Welfare Committee of the University of Veterinary Medicine, Vienna, Austria, and by the Austrian Federal Ministry of Education, Science and Research (permit 2021-0.331.140).

## Author contributions

AB, VMP, RGE, and AW conceived and designed the study. AB and VMP contributed to the acquisition, analysis, and visualization of the data. TK contributed to bioinformatics assistance. AB, VMP, RGE, and AW wrote the manuscript. All authors reviewed and approved the final manuscript. Order of co–senior authors was decided between the two authors.

## Supplementary Material

Supplemental data

## Version 1 10/24/2022

Electronic publication
