# Supplementary material for: Spatial metabolomics reveals upregulation of several pyrophosphate-producing pathways in cortical bone of Hyp mice
Source: JCI Insight. 2022 Oct 24;7(20):e162138. doi: 10.1172/jci.insight.162138 (PMC9714788; doi:10.1172/jci.insight.162138)
Supplement: Supplemental data [file jciinsight-7-162138-s224.pdf]

Supplementary Information for

Spatial metabolomics reveal upregulation of several pyrophosphate-producing pathways in cortical bone of *Hyp* mice

Achim Buck[1]†, Verena M. Prade[1]†, Thomas Kunzke[1], Reinhold G. Erben[2#], Axel Walch[1#]

[1] Research Unit Analytical Pathology, Helmholtz Zentrum München – German Research Center for Environmental Health, Neuherberg, Germany
[2] Department of Biomedical Sciences, University of Veterinary Medicine, Vienna, Austria

† These authors contributed equally to this work
* These authors share the last authorship

*** Correspondence to:**

Axel Walch, MD
Research Unit Analytical Pathology, Helmholtz Zentrum München – German Research Center for Environmental Health, Ingolstädter Landstraße 1, 85764 Neuherberg, Germany
Email: axel.walch@helmholtz-muenchen.de

Reinhold G. Erben, MD, VMD
Department of Biomedical Sciences, University of Veterinary Medicine Vienna, Veterinärplatz 1, 1210 Vienna, Austria
Email: Reinhold.Erben@vetmeduni.ac.at

This pdf file includes:

Figure S1

Figure S2

Table S1

Table S2

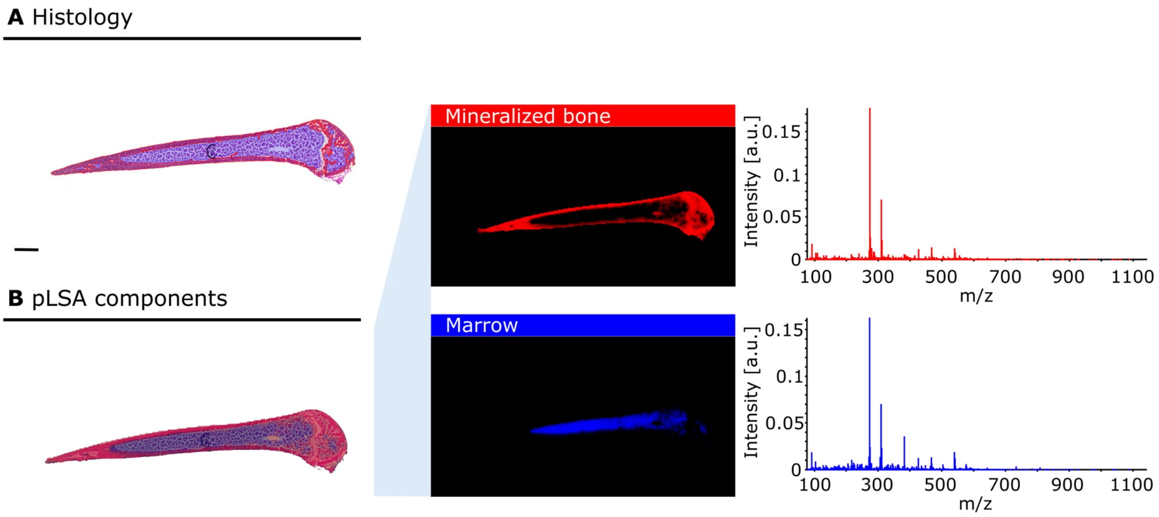

Figure S1. Histology of an undecalcified cryosection of a WT mouse femur and metabolic differences within the bone revealed by MALDI mass spectrometry imaging. **A** HE staining of an undecalcified cryosection of a distal femur from a male 3-month-old WT mouse with annotated mineralized cortical bone (red) and bone marrow (blue). The scanned HE image was annotated in QuPath (v. 0.1.2). **B** Probabilistic latent semantic analysis (pLSA) components generated from spatial metabolomics show differences between the anatomical features and their corresponding characteristic mass spectra on the right. Scale bar = 1 mm.

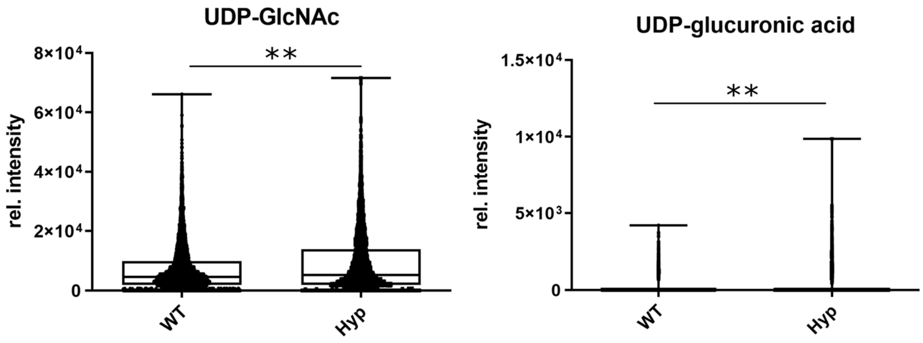

Figure S2. UDP-N-acetyl-glucosamine (UDP-GlcNAc) and UDP-glucuronic acid, both precursors for biosynthesis of glycosaminoglycans and proteoglycans in the extracellular matrix, were significantly more abundant in *Hyp* compared with WT cortical bone (comparison of pixel-wise intensity distribution, n = 5 mice per group). **, P<0.01 by Mann-Whitney test.

Table S1. List of metabolite species altered in *Hyp* cortical bone compared to WT. The analysis is based on mean values (averages) of individuals (n = 5 per group).

m/z	raw p-value	adjusted p-value (Benjamini-Hochberg)	FC	log2(FC)
77.6284	0.0355	0.0533	2.9403	1.5560
79.6312	0.0265	0.0738	7.0335	2.8142
79.7056	0.0109	0.0533	18.1290	4.1803
81.3196	0.0021	0.0304	3.7587	1.9102
81.3227	0.0052	0.0589	19.6240	4.2945
83.6793	0.0116	0.0738	0.2228	-2.1660
85.2059	0.0009	0.0304	16.9680	4.0847
85.6697	0.0023	0.0304	4.1161	2.0413
86.6443	0.0115	0.0304	5.1745	2.3714
86.6475	0.0272	0.0704	4.7846	2.2584
86.6502	0.0231	0.0408	2.6252	1.3924
86.7609	0.0286	0.0704	7.7237	2.9493
88.3181	0.0045	0.0408	10.9380	3.4513
88.3834	0.0267	0.0533	0.2775	-1.8492
89.301	0.0361	0.0738	0.2576	-1.9566
89.6178	0.0201	0.0704	3.3366	1.7384
90.3355	0.0028	0.0304	3.7413	1.9036
91.6677	0.0251	0.0304	0.4292	-1.2204
92.9203	0.0493	0.1066	4.4589	2.1567
92.9685	0.0054	0.0304	3.2041	1.6799
93.586	0.0100	0.0304	5.0230	2.3285
93.9306	0.0160	0.0718	0.3484	-1.5214
94.9916	0.0112	0.0533	2.6438	1.4026
95.6573	0.0144	0.1048	0.2169	-2.2050
95.6603	0.0136	0.0304	3.2937	1.7197
96.9696	0.0227	0.0533	2.1397	1.0974
97.3505	0.0440	0.0704	3.8772	1.9550
97.6069	0.0322	0.0704	0.3522	-1.5055
99.585	0.0221	0.0533	2.3095	1.2076
100.0011	0.0315	0.1066	2.9140	1.5430
102.0266	0.0061	0.0304	9.7825	3.2902
104.2957	0.0049	0.0304	3.4109	1.7701
106.3662	0.0203	0.0704	3.8771	1.9550
108.7292	0.0118	0.0304	0.3866	-1.3712
109.3427	0.0474	0.0704	2.8587	1.5153
110.0318	0.0380	0.0304	3.4428	1.7836
111.9472	0.0476	0.0704	2.1165	1.0817
112.9975	0.0018	0.0304	7.6575	2.9369
114.7038	0.0159	0.0533	6.5713	2.7162

114.9358	0.0140	0.0304	3.3729	1.7540
115.6813	0.0285	0.0408	4.0911	2.0325
116.0157	0.0271	0.0704	8.8423	3.1444
116.9328	0.0066	0.0304	5.4695	2.4514
116.9546	0.0439	0.0704	3.2568	1.7034
117.0284	0.0233	0.1645	2.4978	1.3206
118.0242	0.0393	0.1066	3.2430	1.6973
118.69	0.0488	0.1645	3.1538	1.6571
118.7121	0.0101	0.0304	0.1651	-2.5984
119.6699	0.0089	0.0304	6.3985	2.6777
120.325	0.0037	0.0304	4.0972	2.0346
123.0088	0.0372	0.0533	13.3360	3.7373
124.0086	0.0438	0.0533	2.0314	1.0225
125.0107	0.0490	0.0704	2.0768	1.0544
126.9011	0.0384	0.0704	2.3105	1.2082
127.6738	0.0037	0.0304	40.2570	5.3312
128.3335	0.0264	0.1066	4.8046	2.2644
129.5121	0.0395	0.1066	2.6550	1.4087
132.9368	0.0235	0.0533	4.4054	2.1393
132.9463	0.0020	0.0408	2.9133	1.5427
133.0143	0.0456	0.0533	2.8589	1.5155
134.6733	0.0095	0.0408	3.5038	1.8089
134.9434	0.0015	0.0408	3.5239	1.8172
135.0013	0.0049	0.0304	12.9510	3.6950
135.5019	0.0145	0.0408	6.3242	2.6609
135.503	0.0139	0.0704	8.8322	3.1428
136.9176	0.0044	0.0304	2.0365	1.0261
136.9417	0.0289	0.0533	0.1899	-2.3966
138.9147	0.0018	0.0304	2.6321	1.3962
138.9802	0.0340	0.0704	2.1427	1.0994
142.0094	0.0043	0.0304	3.3359	1.7381
142.0222	0.0057	0.0304	13.9830	3.8056
142.3439	0.0007	0.0304	18.9020	4.2404
143.0301	0.0173	0.0533	15.3980	3.9447
143.4901	0.0381	0.1645	6.3749	2.6724
144.9912	0.0059	0.0304	0.1402	-2.8347
145.5235	0.0355	0.0533	4.8571	2.2801
145.527	0.0124	0.0704	5.5347	2.4685
145.5281	0.0045	0.0304	8.7246	3.1251
146.0141	0.0284	0.0533	4.2342	2.0821
147.0815	0.0489	0.1066	0.3353	-1.5765
148.9646	0.0367	0.0704	5.6189	2.4903
148.9837	0.0008	0.0304	3.0421	1.6051

149.337	0.0289	0.0408	7.9934	2.9988
150.2633	0.0089	0.0506	0.2816	-1.8282
150.9802	0.0140	0.0533	2.1159	1.0813
152.8917	0.0092	0.0304	2.7815	1.4759
152.9193	0.0120	0.0408	0.3311	-1.5946
152.9366	0.0356	0.0533	0.2757	-1.8589
154.8887	0.0092	0.0533	3.5402	1.8238
155.104	0.0465	0.1066	0.2608	-1.9388
156.9253	0.0440	0.0704	2.2071	1.1421
158.9239	0.0002	0.0304	13.1560	3.7176
158.9255	0.0008	0.0304	2.1674	1.1159
158.9269	0.0008	0.0304	11.7160	3.5505
159.0458	0.0256	0.0533	4.5029	2.1709
159.0473	0.0231	0.0533	6.4391	2.6869
159.9841	0.0249	0.0408	3.9262	1.9731
160.9687	0.0089	0.0304	0.1549	-2.6909
161.0456	0.0429	0.1066	2.0778	1.0551
161.5157	0.0274	0.0533	3.9824	1.9937
161.9812	0.0300	0.0408	4.1804	2.0636
163.5105	0.0370	0.0533	3.8659	1.9508
166.0133	0.0033	0.0304	7.7006	2.9450
166.0598	0.0152	0.0533	0.1596	-2.6477
166.4358	0.0290	0.0718	0.3805	-1.3942
166.9752	0.0074	0.0304	5.2842	2.4017
167.0019	0.0232	0.0304	0.2779	-1.8471
167.0211	0.0064	0.0304	3.0625	1.6147
168.6657	0.0002	0.0304	25.4170	4.6677
168.8546	0.0310	0.0304	2.0797	1.0564
168.9143	0.0405	0.1066	0.4724	-1.0819
168.9908	0.0026	0.0304	8.6373	3.1106
169.0184	0.0371	0.0533	0.1961	-2.3507
169.9059	0.0221	0.0533	2.6181	1.3885
170.9022	0.0314	0.0533	2.5532	1.3523
170.9112	0.0477	0.1066	0.4638	-1.1086
171.0065	0.0212	0.0533	2.1631	1.1131
172.8944	0.0070	0.0408	4.8023	2.2637
172.8992	0.0409	0.0533	2.7755	1.4728
172.9914	0.0109	0.0304	5.9791	2.5799
173.5198	0.0143	0.0408	5.9682	2.5773
173.5215	0.0127	0.0533	7.8388	2.9706
173.8827	0.0178	0.0533	3.0330	1.6008
174.8914	0.0059	0.0408	5.2626	2.3958
174.9569	0.0014	0.0304	14.5930	3.8672

175.9885	0.0063	0.0304	7.2095	2.8499
175.9933	0.0205	0.0408	4.8137	2.2671
176.9265	0.0094	0.0304	5.0311	2.3309
176.9336	0.0346	0.0408	4.9405	2.3047
176.936	0.0128	0.0304	2.1338	1.0934
176.938	0.0248	0.1066	3.2778	1.7127
176.9637	0.0307	0.0533	0.2611	-1.9372
177.9855	0.0023	0.0408	12.1400	3.6017
178.8445	0.0254	0.1048	0.3313	-1.5936
180.05	0.0039	0.0304	9.3607	3.2266
180.3779	0.0417	0.1066	6.3049	2.6565
180.9073	0.0030	0.0408	4.4565	2.1559
181.0289	0.0417	0.0408	2.1124	1.0789
181.9661	0.0387	0.0533	2.2114	1.1450
181.9685	0.0411	0.0533	3.0192	1.5941
182.0225	0.0153	0.0533	5.0381	2.3329
182.337	0.0166	0.0533	0.4561	-1.1326
182.9654	0.0486	0.0704	3.8974	1.9625
183.963	0.0439	0.0533	2.2412	1.1643
184.02	0.0134	0.0533	6.5351	2.7082
184.5127	0.0267	0.0533	2.3900	1.2570
184.9857	0.0015	0.0304	19.8850	4.3136
185.0079	0.0186	0.0408	5.0801	2.3449
185.9632	0.0194	0.0704	3.4398	1.7823
187.0028	0.0277	0.0533	8.7748	3.1334
188.9725	0.0411	0.0533	4.6628	2.2212
190.8924	0.0006	0.0304	2.3641	1.2413
190.905	0.0198	0.0533	4.0685	2.0245
190.9695	0.0085	0.0304	5.4116	2.4361
191.016	0.0062	0.0408	11.5340	3.5278
191.0198	0.0170	0.0304	2.9545	1.5629
191.5733	0.0112	0.0304	3.6367	1.8626
191.8932	0.0003	0.0304	5.5836	2.4812
192.8893	0.0025	0.0304	2.4875	1.3147
192.902	0.0263	0.0533	4.1567	2.0554
192.9586	0.0196	0.0408	0.2155	-2.2144
193.0366	0.0243	0.0408	2.9893	1.5798
194.8867	0.0294	0.0704	2.5590	1.3556
194.902	0.0075	0.0408	6.9969	2.8067
194.9466	0.0007	0.0304	10.6460	3.4123
196.0285	0.0406	0.0533	2.6907	1.4280
196.9389	0.0004	0.0304	4.7950	2.2615
197.94	0.0123	0.0533	2.3773	1.2493

197.9424	0.0310	0.0533	3.6107	1.8523
197.9706	0.0085	0.0304	4.2316	2.0812
198.9082	0.0334	0.0704	3.2053	1.6804
198.9432	0.0435	0.0533	3.3512	1.7447
199.1129	0.0256	0.0533	0.1466	-2.7697
199.9371	0.0088	0.0533	2.4034	1.2651
199.9389	0.0225	0.0533	2.9659	1.5685
199.9674	0.0206	0.0704	5.9852	2.5814
200.0563	0.0449	0.0704	3.8105	1.9300
200.936	0.0443	0.0704	2.2483	1.1688
201.9329	0.0399	0.1066	3.4135	1.7712
201.935	0.0267	0.0533	3.4781	1.7983
201.9676	0.0050	0.0408	4.0612	2.0219
201.9942	0.0067	0.0304	11.4570	3.5182
202.9519	0.0236	0.0533	4.0667	2.0239
202.9657	0.0485	0.1066	5.5277	2.4667
202.9791	0.0480	0.0304	0.1889	-2.4045
202.9978	0.0012	0.0304	19.0910	4.2548
203.9738	0.0350	0.0533	5.7725	2.5292
204.0052	0.0024	0.0304	5.3070	2.4079
205.062	0.0036	0.0304	0.1508	-2.7296
206.8788	0.0036	0.0304	7.1937	2.8467
206.9678	0.0266	0.0408	3.6895	1.8834
206.9831	0.0039	0.0304	9.8852	3.3053
207.0803	0.0306	0.0704	0.0808	-3.6300
207.0928	0.0144	0.0533	0.0757	-3.7234
207.9815	0.0223	0.0533	2.5480	1.3493
208.0882	0.0415	0.0704	0.0969	-3.3681
208.8759	0.0032	0.0304	7.0286	2.8132
208.9802	0.0012	0.0304	10.7540	3.4268
208.9849	0.0021	0.0304	2.8845	1.5283
209.0677	0.0442	0.0704	0.2008	-2.3164
209.94	0.0321	0.0533	3.1296	1.6460
210.0521	0.0193	0.0704	0.2703	-1.8872
210.0561	0.0455	0.0704	0.2042	-2.2919
210.0997	0.0419	0.0704	0.3516	-1.5081
210.1249	0.0255	0.1066	0.2139	-2.2252
210.9986	0.0329	0.1066	2.6946	1.4301
211.9369	0.0114	0.0533	4.4323	2.1481
211.9652	0.0144	0.0533	5.4645	2.4501
212.9127	0.0394	0.0704	3.5896	1.8438
213.0111	0.0043	0.0304	7.8983	2.9815
213.0134	0.0029	0.0304	9.9243	3.3110

213.0172	0.0042	0.0304	2.9635	1.5673
213.0993	0.0270	0.0704	0.1909	-2.3892
213.9444	0.0391	0.0533	3.7995	1.9258
215.0328	0.0002	0.0304	2.8263	1.4989
215.115	0.0144	0.0533	0.1636	-2.6114
215.9975	0.0444	0.1066	0.2601	-1.9430
216.0362	0.0070	0.0408	4.5475	2.1851
216.8842	0.0332	0.1066	4.8585	2.2805
217.0302	0.0014	0.0304	5.6585	2.5004
217.0772	0.0384	0.0704	0.1113	-3.1670
218.0459	0.0202	0.0408	0.0081	-6.9514
218.9467	0.0253	0.0533	4.4795	2.1633
218.9969	0.0325	0.1590	0.3838	-1.3817
219.0563	0.0335	0.0533	0.1705	-2.5522
220.7766	0.0178	0.0506	0.1990	-2.3289
220.9622	0.0089	0.0533	7.8971	2.9813
221.9748	0.0059	0.0304	2.5949	1.3757
221.9843	0.0099	0.0704	5.3686	2.4245
222.0755	0.0227	0.0408	0.1190	-3.0710
222.0996	0.0062	0.0304	0.1485	-2.7515
223.0646	0.0071	0.0304	0.1536	-2.7029
223.9557	0.0269	0.0408	2.3618	1.2399
224.0548	0.0493	0.0704	0.2303	-2.1184
224.9798	0.0039	0.0304	7.0136	2.8102
225.0628	0.0450	0.1066	0.1552	-2.6880
225.1499	0.0342	0.0408	0.0916	-3.4479
226.051	0.0436	0.0704	0.1339	-2.9007
226.9496	0.0036	0.0304	3.4380	1.7816
227.9134	0.0043	0.0304	4.6942	2.2309
228.9662	0.0104	0.0408	2.6862	1.4256
229.0119	0.0136	0.0304	2.3627	1.2404
229.032	0.0171	0.0408	0.1531	-2.7075
229.142	0.0310	0.0704	0.2082	-2.2641
230.8488	0.0404	0.0704	2.8187	1.4950
230.8579	0.0207	0.0304	2.8838	1.5280
230.9501	0.0001	0.0304	23.9910	4.5844
230.9621	0.0011	0.0304	7.3656	2.8808
231.9817	0.0416	0.0704	3.6150	1.8540
232.8802	0.0352	0.0533	4.2718	2.0949
232.8835	0.0135	0.0408	2.1242	1.0869
232.8931	0.0051	0.0304	6.3782	2.6731
232.9028	0.0069	0.0408	4.5679	2.1915
232.9472	0.0000	0.0304	8.4538	3.0796

232.9835	0.0257	0.0533	5.8793	2.5557
233.1086	0.0070	0.0408	0.0820	-3.6079
233.9166	0.0220	0.0408	4.2688	2.0938
233.9346	0.0207	0.0533	7.1983	2.8477
234.125	0.0443	0.0704	0.3114	-1.6832
234.8853	0.0000	0.0304	14.2900	3.8369
234.8945	0.0438	0.1066	3.0880	1.6267
234.9781	0.0067	0.0304	7.3500	2.8777
235.9137	0.0269	0.0408	4.3154	2.1095
236.7716	0.0373	0.1066	4.0513	2.0184
236.8916	0.0495	0.0704	5.1530	2.3654
237.1134	0.0349	0.1645	0.2719	-1.8791
237.1498	0.0366	0.0408	0.1470	-2.7663
237.9924	0.0402	0.0704	2.3578	1.2374
238.8767	0.0150	0.0533	3.9623	1.9863
238.9304	0.0101	0.0408	0.3390	-1.5607
239.0343	0.0290	0.0704	4.0944	2.0337
239.0694	0.0387	0.0704	0.2962	-1.7552
239.9967	0.0285	0.0533	4.2844	2.0991
241.0662	0.0381	0.0408	0.2676	-1.9017
241.9411	0.0320	0.0704	2.3632	1.2408
241.9476	0.0440	0.0704	3.7165	1.8939
242.7903	0.0263	0.0704	3.4475	1.7855
242.9444	0.0092	0.0304	9.0974	3.1854
243.1099	0.0169	0.0533	0.2617	-1.9338
243.1828	0.0226	0.0408	0.2890	-1.7907
243.9192	0.0242	0.0704	2.4798	1.3102
243.957	0.0007	0.0304	2.1383	1.0965
243.9606	0.0051	0.0304	4.6207	2.2081
243.9664	0.0021	0.0304	3.5526	1.8289
243.9897	0.0042	0.0408	2.5468	1.3487
244.0173	0.0489	0.0704	0.1638	-2.6101
244.7874	0.0380	0.0704	3.4618	1.7915
244.939	0.0049	0.0408	4.8174	2.2683
244.9599	0.0084	0.0408	3.3890	1.7608
244.9626	0.0134	0.0533	3.2331	1.6929
245.0125	0.0210	0.0704	12.4750	3.6409
245.9527	0.0018	0.0304	3.2847	1.7158
246.0142	0.0287	0.0304	0.3827	-1.3856
246.8232	0.0076	0.0304	3.1274	1.6450
246.9363	0.0197	0.0408	5.7121	2.5140
246.9781	0.0490	0.0533	3.0261	1.5975
247.0363	0.0327	0.0533	2.0211	1.0152

247.1191	0.0101	0.0533	0.2121	-2.2370
248.8638	0.0240	0.0533	2.5469	1.3487
248.9393	0.0118	0.0408	6.5450	2.7104
248.9572	0.0158	0.0408	4.7984	2.2626
248.9784	0.0064	0.0304	5.1070	2.3525
249.0223	0.0044	0.0408	3.2581	1.7040
249.1037	0.0254	0.1066	0.1059	-3.2396
250.0181	0.0144	0.0408	0.2468	-2.0188
250.0257	0.0294	0.0704	3.7204	1.8955
250.8333	0.0059	0.0304	2.2883	1.1943
250.9244	0.0206	0.0568	0.2510	-1.9941
252.8305	0.0031	0.0304	2.4865	1.3141
252.9939	0.0033	0.0304	15.6690	3.9699
253.9874	0.0254	0.0704	6.9634	2.7998
254.7644	0.0175	0.0533	3.2282	1.6907
254.8867	0.0455	0.0589	0.4232	-1.2404
255.1826	0.0466	0.0718	0.1737	-2.5257
255.8984	0.0055	0.0304	2.8870	1.5296
256.8672	0.0192	0.0304	6.5046	2.7015
256.9022	0.0223	0.0704	2.8483	1.5101
257.0069	0.0084	0.0304	3.1190	1.6411
257.1256	0.0136	0.0533	0.2508	-1.9955
257.8956	0.0097	0.0304	3.2642	1.7068
258.0603	0.0408	0.0704	0.4230	-1.2412
258.951	0.0282	0.0568	0.3122	-1.6796
258.992	0.0121	0.0533	6.3098	2.6576
259.9308	0.0015	0.0304	2.4918	1.3172
259.9404	0.0136	0.0304	3.1875	1.6724
259.9767	0.0298	0.0533	2.5089	1.3270
260.0713	0.0346	0.0533	2.2901	1.1954
261.0075	0.0124	0.0533	9.2569	3.2105
261.1036	0.0346	0.1645	0.1105	-3.1779
261.9263	0.0074	0.0304	4.6765	2.2254
261.9385	0.0358	0.0704	2.2991	1.2011
262.0869	0.0109	0.0408	2.3219	1.2153
263.05	0.0054	0.0304	3.6915	1.8842
263.9716	0.0209	0.1066	2.4648	1.3015
264.8191	0.0285	0.1066	3.3696	1.7526
264.9526	0.0053	0.0533	7.1326	2.8344
264.9887	0.0000	0.0304	12.6000	3.6554
265.0443	0.0233	0.0704	2.6783	1.4213
265.0849	0.0159	0.0533	0.1209	-3.0478
265.1309	0.0245	0.1066	0.3244	-1.6241

265.8845	0.0321	0.0304	3.7043	1.8892
265.9047	0.0302	0.0533	6.0161	2.5888
265.9873	0.0233	0.0533	3.2772	1.7125
266.1149	0.0183	0.0533	0.2878	-1.7970
266.816	0.0113	0.0533	4.3428	2.1186
266.9097	0.0175	0.0704	0.2330	-2.1018
268.0126	0.0333	0.0704	2.2510	1.1706
268.9882	0.0008	0.0304	5.6086	2.4876
269.1732	0.0184	0.0589	0.2613	-1.9361
269.982	0.0283	0.0704	2.4490	1.2922
270.939	0.0266	0.0304	4.3267	2.1133
270.9878	0.0348	0.0704	0.3763	-1.4101
270.9997	0.0108	0.0408	7.7937	2.9623
271.0041	0.0010	0.0304	2.7140	1.4404
271.0086	0.0014	0.0304	7.7581	2.9557
271.0608	0.0103	0.0408	6.2640	2.6471
271.1413	0.0153	0.0408	0.2218	-2.1728
271.8726	0.0235	0.0533	3.5840	1.8416
272.0077	0.0013	0.0304	4.2416	2.0846
272.0889	0.0035	0.0304	3.3909	1.7617
272.9999	0.0072	0.0408	3.8187	1.9331
273.0077	0.0365	0.0533	2.1070	1.0752
273.0918	0.0305	0.0533	2.6586	1.4107
273.1343	0.0058	0.0408	0.1312	-2.9297
273.8697	0.0471	0.0704	3.9603	1.9856
275.8805	0.0324	0.0533	4.0130	2.0047
276.0991	0.0280	0.1066	0.3493	-1.5174
278.5411	0.0205	0.0533	3.2754	1.7117
278.8843	0.0319	0.0533	6.0509	2.5971
278.9115	0.0451	0.0533	0.3649	-1.4543
279.9306	0.0425	0.1066	5.1872	2.3750
279.9427	0.0110	0.0533	6.2427	2.6422
279.9664	0.0132	0.0408	3.0384	1.6033
280.7552	0.0411	0.0704	2.0746	1.0528
280.9223	0.0297	0.0533	3.8619	1.9493
281.8972	0.0288	0.0718	0.3884	-1.3645
281.9824	0.0254	0.0704	4.5665	2.1911
282.125	0.0028	0.0304	8.1104	3.0198
282.7731	0.0002	0.0304	2.7486	1.4587
282.9096	0.0000	0.0304	0.1170	-3.0951
282.9289	0.0267	0.0533	2.2754	1.1861
282.9311	0.0145	0.0568	0.3100	-1.6899
283.0037	0.0023	0.0408	4.7799	2.2570

283.1016	0.0367	0.0533	3.8365	1.9398
283.1086	0.0065	0.0533	5.1425	2.3625
283.7813	0.0146	0.0568	0.2297	-2.1220
284.0931	0.0101	0.0533	5.9512	2.5732
284.7495	0.0010	0.0304	3.3084	1.7262
284.9547	0.0289	0.0704	2.5925	1.3743
284.9631	0.0079	0.0304	3.9686	1.9886
285.0485	0.0473	0.1066	6.2848	2.6519
285.0924	0.0408	0.0533	0.1272	-2.9754
285.1205	0.0265	0.0533	0.2007	-2.3168
286.0603	0.0459	0.0704	3.1612	1.6605
286.9338	0.0182	0.0533	7.9771	2.9959
286.9783	0.0015	0.0304	2.7248	1.4462
286.983	0.0121	0.0533	7.5948	2.9250
287.0633	0.0364	0.0704	6.4929	2.6989
287.1191	0.0447	0.0704	0.0742	-3.7524
287.8061	0.0427	0.0304	0.4628	-1.1115
287.9811	0.0079	0.0304	4.4060	2.1395
288.9732	0.0170	0.0304	3.7988	1.9255
288.9771	0.0110	0.0304	4.5608	2.1893
289.0329	0.0007	0.0304	10.9290	3.4501
290.0227	0.0078	0.0533	0.0801	-3.6412
290.1148	0.0435	0.0533	0.2828	-1.8224
290.8426	0.0400	0.0533	2.1453	1.1012
291.0387	0.0433	0.0533	3.7787	1.9179
291.0492	0.0252	0.0533	5.0649	2.3405
291.1004	0.0280	0.0304	0.1258	-2.9910
291.1462	0.0251	0.0704	0.2044	-2.2902
291.8666	0.0277	0.0533	2.7884	1.4794
291.8751	0.0200	0.0533	3.7489	1.9065
292.0337	0.0003	0.0304	7.2826	2.8645
292.9413	0.0010	0.0304	11.0910	3.4713
292.9836	0.0359	0.0704	2.3917	1.2581
293.8638	0.0255	0.0533	2.7641	1.4668
293.8725	0.0241	0.0704	4.0717	2.0256
294.0582	0.0239	0.0533	3.5783	1.8393
294.9992	0.0248	0.0704	4.1906	2.0672
295.861	0.0104	0.0408	3.0554	1.6114
295.908	0.0434	0.1066	2.6417	1.4014
295.9168	0.0401	0.0533	5.3177	2.4108
296.008	0.0111	0.0533	5.4355	2.4424
296.048	0.0110	0.0533	6.3829	2.6742
296.1004	0.0453	0.1590	0.2382	-2.0700

296.9961	0.0130	0.0704	4.0214	2.0077
297.0195	0.0132	0.0304	5.4878	2.4562
297.1206	0.0242	0.0533	0.2869	-1.8014
297.9045	0.0278	0.0533	2.6126	1.3855
298.0452	0.0063	0.0408	5.3956	2.4318
298.0637	0.0132	0.0533	5.8651	2.5521
298.7516	0.0155	0.0408	2.4750	1.3074
298.8472	0.0449	0.0704	0.3331	-1.5861
298.9786	0.0023	0.0408	3.7971	1.9249
298.999	0.0321	0.1066	2.5081	1.3266
299.1191	0.0415	0.1066	0.3631	-1.4616
299.1726	0.0203	0.0533	0.2317	-2.1095
300.179	0.0129	0.0533	0.1883	-2.4087
300.7488	0.0128	0.0408	2.4332	1.2829
300.8734	0.0251	0.0408	2.3103	1.2080
301.9158	0.0428	0.1645	2.3761	1.2486
301.9415	0.0479	0.0533	3.3204	1.7314
302.7462	0.0159	0.0408	2.5660	1.3595
302.7751	0.0072	0.0304	5.9334	2.5689
302.9081	0.0393	0.0704	5.4286	2.4406
303.9703	0.0335	0.1066	4.1165	2.0414
303.9895	0.0267	0.0704	4.1820	2.0642
304.0112	0.0055	0.0408	5.2728	2.3986
304.0606	0.0031	0.0304	4.1673	2.0591
304.0708	0.0153	0.0704	5.4544	2.4474
304.7721	0.0061	0.0304	6.3487	2.6665
304.9834	0.0472	0.0704	3.3525	1.7452
306.0768	0.0009	0.0304	2.6623	1.4127
306.9809	0.0299	0.0533	4.9232	2.2996
307.08	0.0085	0.0408	6.9714	2.8014
308.0722	0.0015	0.0408	6.9310	2.7931
308.7313	0.0050	0.0408	3.3529	1.7454
309.1206	0.0219	0.0704	0.3530	-1.5024
310.7891	0.0355	0.0408	2.0366	1.0262
312.1384	0.0028	0.0453	0.2960	-1.7562
312.7861	0.0331	0.0408	2.9304	1.5511
312.9942	0.0123	0.0533	3.6923	1.8845
313.0142	0.0040	0.0304	10.4780	3.3893
313.1347	0.0332	0.0738	0.1383	-2.8547
314.1473	0.0047	0.0408	0.2328	-2.1030
314.7255	0.0301	0.0533	2.4995	1.3216
314.9564	0.0222	0.0533	2.7595	1.4644
314.9658	0.0011	0.0304	3.7584	1.9101

315.8542	0.0225	0.0533	3.7364	1.9017
316.7231	0.0105	0.0533	2.4901	1.3162
316.7318	0.0164	0.0533	0.0460	-4.4421
316.9813	0.0009	0.0304	4.6043	2.2030
317.1615	0.0136	0.0704	0.1461	-2.7752
318.003	0.0019	0.0304	5.6574	2.5001
318.0831	0.0304	0.0704	6.4907	2.6984
318.0858	0.0241	0.0533	4.5224	2.1771
318.0919	0.0292	0.0408	2.7532	1.4611
318.098	0.0280	0.0533	5.0209	2.3279
318.7199	0.0123	0.0533	2.9072	1.5396
318.9182	0.0365	0.1048	0.0785	-3.6718
318.9786	0.0146	0.0304	4.7556	2.2496
319.0395	0.0174	0.0718	0.3246	-1.6232
319.0692	0.0122	0.1066	2.6751	1.4196
319.0955	0.0441	0.0408	2.8810	1.5266
319.9885	0.0275	0.0704	5.1412	2.3621
320.0796	0.0481	0.1066	2.7668	1.4682
320.0872	0.0487	0.0533	3.4351	1.7804
320.0986	0.0128	0.0533	3.8176	1.9327
320.7172	0.0335	0.0704	3.3694	1.7525
321.091	0.0281	0.0704	3.3063	1.7252
322.0373	0.0247	0.0533	9.8166	3.2952
322.908	0.0079	0.0568	0.1743	-2.5200
322.955	0.0115	0.0533	7.9181	2.9852
323.9102	0.0045	0.0304	3.8325	1.9383
324.0692	0.0368	0.0533	2.5733	1.3636
324.7663	0.0055	0.0304	4.9993	2.3217
325.1655	0.0177	0.0304	0.1763	-2.5041
325.2017	0.0250	0.0568	0.2778	-1.8479
326.0083	0.0218	0.0533	9.1273	3.1902
326.0429	0.0451	0.0533	5.4137	2.4366
326.1589	0.0364	0.0533	0.1873	-2.4168
326.196	0.0279	0.0704	0.2600	-1.9436
326.7633	0.0063	0.0408	5.5788	2.4800
326.8454	0.0197	0.0568	0.2967	-1.7531
326.9136	0.0372	0.0589	0.3657	-1.4513
327.0033	0.0052	0.0304	2.5159	1.3310
328.0148	0.0480	0.0704	2.7445	1.4566
328.0351	0.0099	0.0408	7.4586	2.8989
328.163	0.0411	0.0704	0.2354	-2.0867
328.2104	0.0222	0.0408	0.1547	-2.6926
328.9629	0.0247	0.0533	4.3034	2.1055

328.9891	0.0104	0.0304	5.0641	2.3403
329.029	0.0427	0.0704	3.1815	1.6697
329.8313	0.0045	0.0304	3.5799	1.8399
330.0217	0.0457	0.0533	3.1428	1.6520
330.092	0.0444	0.0304	2.4401	1.2870
330.9601	0.0028	0.0408	4.6265	2.2099
331.0138	0.0426	0.0704	3.4550	1.7887
331.0951	0.0343	0.0304	3.5911	1.8444
331.8288	0.0310	0.0533	3.4743	1.7967
332.6965	0.0136	0.0704	4.3054	2.1061
332.9549	0.0000	0.0304	7.4435	2.8960
333.0644	0.0138	0.0408	6.3632	2.6697
333.1926	0.0271	0.0704	0.1360	-2.8783
333.9771	0.0062	0.0408	4.9168	2.2977
334.0963	0.0033	0.0304	5.3569	2.4214
334.6939	0.0157	0.0533	4.1732	2.0612
334.9521	0.0036	0.0304	2.9858	1.5781
335.1719	0.0138	0.0704	0.2391	-2.0646
336.1747	0.0093	0.0408	0.1663	-2.5880
336.6915	0.0197	0.0704	3.8895	1.9596
336.9734	0.0117	0.0304	5.1852	2.3744
338.0488	0.0123	0.0408	8.5251	3.0917
338.8857	0.0081	0.0568	0.2132	-2.2297
338.9895	0.0071	0.0304	3.4529	1.7878
339.992	0.0047	0.0304	4.4794	2.1633
342.1785	0.0087	0.0533	0.2261	-2.1450
343.1768	0.0058	0.0304	0.0932	-3.4228
343.961	0.0430	0.1066	3.1183	1.6407
344.0004	0.0215	0.0533	9.6302	3.2676
344.0186	0.0280	0.0704	7.0735	2.8224
344.0329	0.0014	0.0304	7.1267	2.8332
344.1081	0.0278	0.0408	3.1643	1.6619
344.8507	0.0049	0.0533	0.1501	-2.7362
344.9365	0.0411	0.1066	4.5006	2.1701
344.9549	0.0402	0.1066	2.8501	1.5110
345.0244	0.0244	0.0533	5.3168	2.4106
345.1108	0.0200	0.0533	5.1878	2.3751
346.0486	0.0091	0.0533	2.4627	1.3003
346.775	0.0013	0.0304	3.3944	1.7631
346.9341	0.0392	0.0704	3.5899	1.8439
346.9843	0.0293	0.0704	3.6579	1.8710
347.0295	0.0313	0.0704	9.4702	3.2434
347.0328	0.0032	0.0408	10.6850	3.4176

347.04	0.0154	0.0408	3.2722	1.7103
347.0473	0.0048	0.0533	11.1240	3.4756
347.0518	0.0249	0.0704	7.6623	2.9378
347.0667	0.0438	0.0704	2.5047	1.3246
347.8027	0.0287	0.0533	4.2933	2.1021
347.979	0.0022	0.0304	6.8546	2.7771
348.0437	0.0218	0.0304	3.9936	1.9977
348.9141	0.0283	0.0704	5.4144	2.4368
349.0442	0.0070	0.0704	9.9144	3.3095
349.0503	0.0090	0.0408	0.0381	-4.7128
351.0985	0.0038	0.0304	8.3815	3.0672
352.0533	0.0476	0.0533	2.9789	1.5748
352.6945	0.0003	0.0371	0.1242	-3.0095
354.0406	0.0422	0.1066	5.0834	2.3458
354.0499	0.0158	0.0704	3.5593	1.8316
354.076	0.0269	0.1066	4.7962	2.2619
355.0721	0.0461	0.1066	3.1883	1.6728
355.8973	0.0035	0.0304	11.2360	3.4900
357.0552	0.0048	0.0304	2.5515	1.3513
357.0693	0.0380	0.1066	3.2257	1.6896
357.998	0.0052	0.0408	3.7662	1.9131
358.0208	0.0250	0.0533	4.8728	2.2848
358.1232	0.0143	0.0408	4.5889	2.1982
359.0051	0.0305	0.0304	3.2257	1.6896
360.0087	0.0130	0.0408	6.0450	2.5958
360.1032	0.0170	0.0408	4.7147	2.2372
360.9708	0.0025	0.0304	3.5075	1.8104
361.0027	0.0137	0.0304	5.2806	2.4007
361.0867	0.0278	0.0533	3.9742	1.9907
361.187	0.0109	0.0533	0.1897	-2.3983
361.974	0.0041	0.0408	3.4007	1.7658
362.0431	0.0391	0.0704	4.7125	2.2365
364.0107	0.0050	0.0408	6.4138	2.6812
364.0218	0.0236	0.0704	3.0693	1.6179
364.0356	0.0154	0.0304	3.7496	1.9067
364.1095	0.0349	0.0589	0.3322	-1.5897
364.7569	0.0049	0.0408	3.3085	1.7262
366.0191	0.0156	0.0533	4.2355	2.0825
366.0268	0.0025	0.0304	24.3080	4.6034
366.1897	0.0295	0.0704	0.2626	-1.9293
367.2099	0.0225	0.0533	0.1788	-2.4838
368.0657	0.0206	0.0533	3.2272	1.6903
368.1572	0.0204	0.0304	0.3030	-1.7227

368.8246	0.0032	0.0304	4.4776	2.1627
369.0224	0.0145	0.0304	4.8199	2.2690
370.025	0.0491	0.0704	11.0660	3.4681
370.7445	0.0145	0.0408	2.1576	1.1094
370.9338	0.0006	0.0304	12.9800	3.6982
372.8272	0.0130	0.0506	0.1742	-2.5215
372.8953	0.0004	0.0304	4.5731	2.1932
373.813	0.0458	0.0704	2.5481	1.3494
375.0486	0.0357	0.0589	0.3476	-1.5247
376.0937	0.0327	0.0589	3.4726	1.7960
376.6787	0.0117	0.0533	2.6479	1.4049
376.9195	0.0081	0.0408	4.8585	2.2805
376.9443	0.0064	0.0304	3.8976	1.9626
378.9169	0.0232	0.0533	4.3011	2.1047
379.1983	0.0281	0.1066	0.2267	-2.1414
380.0843	0.0370	0.0704	3.3715	1.7534
380.2007	0.0160	0.0704	0.1107	-3.1754
380.8107	0.0059	0.0304	2.3437	1.2288
380.9569	0.0322	0.0738	0.2756	-1.8596
380.9871	0.0129	0.0304	2.2236	1.1529
382.0235	0.0267	0.0704	6.4873	2.6976
382.0323	0.0280	0.0408	2.7992	1.4850
382.0813	0.0460	0.1066	2.8224	1.4969
382.1735	0.0341	0.0533	0.2358	-2.0846
382.9848	0.0267	0.0408	3.6039	1.8496
383.0158	0.0012	0.0304	9.9432	3.3137
383.036	0.0410	0.0533	3.2036	1.6797
384.0202	0.0021	0.0304	26.2910	4.7165
384.0603	0.0116	0.1066	2.3012	1.2024
384.7092	0.0003	0.0304	3.7804	1.9186
384.7221	0.0021	0.0304	2.3987	1.2622
384.8106	0.0127	0.0568	0.2157	-2.2132
384.9956	0.0168	0.0304	5.6442	2.4968
385.0135	0.0006	0.0304	18.3850	4.2005
385.9934	0.0127	0.0304	48.0580	5.5867
386.076	0.0446	0.0533	5.4565	2.4480
386.1666	0.0262	0.0533	0.1736	-2.5264
386.9939	0.0321	0.0704	5.1082	2.3528
387.2032	0.0143	0.0704	0.1261	-2.9872
387.79	0.0073	0.0408	3.1780	1.6681
388.0092	0.0002	0.0304	26.8670	4.7478
388.6583	0.0241	0.0704	3.6573	1.8708
389.7869	0.0108	0.0408	2.9975	1.5837

391.7839	0.0185	0.0533	3.1828	1.6703
392.6526	0.0171	0.0304	3.7996	1.9259
393.2135	0.0239	0.0704	0.2249	-2.1529
394.1845	0.0318	0.0408	0.3681	-1.4419
395.2285	0.0043	0.0304	0.1105	-3.1780
395.6837	0.0371	0.0533	3.2412	1.6965
396.9477	0.0003	0.0304	11.5290	3.5272
397.0773	0.0406	0.0704	2.7188	1.4430
398.0266	0.0051	0.0408	5.7065	2.5126
398.0761	0.0151	0.0408	3.7342	1.9008
399.1611	0.0294	0.0704	0.1827	-2.4523
400.0909	0.0279	0.0704	11.0490	3.4658
400.9153	0.0463	0.2384	2.5624	1.3575
401.1318	0.0444	0.0704	0.1209	-3.0480
401.1599	0.0298	0.0704	0.1978	-2.3380
402.0105	0.0304	0.0533	3.1869	1.6721
402.9541	0.0061	0.0304	8.6404	3.1111
402.9852	0.0484	0.0704	2.4644	1.3012
403.234	0.0351	0.0704	0.1127	-3.1498
403.9832	0.0010	0.0304	12.6330	3.6592
404.015	0.0004	0.0304	2.0134	1.0096
404.9868	0.0194	0.0533	5.6085	2.4876
404.9989	0.0018	0.0304	3.8311	1.9378
405.0176	0.0048	0.0408	2.9003	1.5362
405.0859	0.0444	0.1066	3.1720	1.6654
405.2138	0.0163	0.1066	0.1796	-2.4769
405.7614	0.0309	0.0704	4.2557	2.0894
406.0008	0.0081	0.0304	4.7527	2.2487
406.0114	0.0007	0.0304	2.2859	1.1928
406.2162	0.0264	0.0408	0.2495	-2.0030
406.9833	0.0374	0.0704	3.7135	1.8928
406.9972	0.0012	0.0304	12.6400	3.6599
408.0007	0.0040	0.0304	5.8351	2.5448
408.0214	0.0349	0.0304	5.5884	2.4824
409.1839	0.0496	0.0704	0.3305	-1.5972
410.0159	0.0084	0.0533	3.7714	1.9151
410.205	0.0442	0.0738	0.1983	-2.3343
412.0265	0.0380	0.0704	3.1819	1.6699
412.1645	0.0241	0.1048	0.3125	-1.6779
412.6144	0.0140	0.0568	0.2976	-1.7486
412.921	0.0089	0.0408	5.7685	2.5282
413.0304	0.0252	0.0533	4.1003	2.0357
414.0242	0.0410	0.1066	3.1073	1.6357

414.8852	0.0376	0.0704	2.1847	1.1274
414.8921	0.0151	0.0408	4.2442	2.0855
416.129	0.0493	0.1066	0.0970	-3.3661
416.8903	0.0441	0.0533	3.0804	1.6231
419.9881	0.0150	0.0408	2.5158	1.3310
420.0094	0.0075	0.0304	3.6098	1.8519
420.9634	0.0056	0.0408	6.4336	2.6856
420.9717	0.0034	0.0304	14.2890	3.8369
421.245	0.0058	0.0408	0.1049	-3.2527
421.9862	0.0486	0.0704	2.8508	1.5114
422.0071	0.0059	0.0408	3.2506	1.7007
422.9611	0.0115	0.0408	8.1811	3.0323
422.9689	0.0489	0.0704	11.8870	3.5713
423.1997	0.0420	0.0533	0.2617	-1.9340
423.2238	0.0255	0.0704	0.2467	-2.0195
424.1845	0.0351	0.0533	0.2433	-2.0391
426.0076	0.0028	0.0304	8.7782	3.1339
426.0119	0.0022	0.0304	10.5810	3.4034
426.0222	0.0011	0.0304	2.9001	1.5361
426.0332	0.0015	0.0304	10.0860	3.3343
426.0375	0.0032	0.0408	12.4310	3.6359
426.9804	0.0293	0.0304	4.2625	2.0917
427.0067	0.0092	0.0408	16.6030	4.0533
427.0165	0.0001	0.0304	6.1438	2.6191
427.0257	0.0072	0.0304	2.6587	1.4107
427.0588	0.0500	0.0738	0.3584	-1.4803
428.0007	0.0215	0.0533	6.4688	2.6935
428.9778	0.0104	0.0408	9.2451	3.2087
431.2279	0.0126	0.0533	0.1471	-2.7654
432.2316	0.0151	0.0304	0.3303	-1.5981
433.0567	0.0405	0.0704	14.9050	3.8977
435.9833	0.0064	0.0408	5.6369	2.4949
436.6346	0.0014	0.0304	2.8776	1.5249
437.2397	0.0352	0.0408	0.2703	-1.8874
439.2547	0.0394	0.0704	0.1929	-2.3740
442.9783	0.0161	0.0304	5.4564	2.4479
443.1872	0.0405	0.0533	0.1499	-2.7382
446.6176	0.0448	0.0704	2.4421	1.2881
447.26	0.0227	0.0704	0.0999	-3.3227
448.0047	0.0258	0.0408	2.1511	1.1051
448.0162	0.0377	0.1066	3.9492	1.9816
448.2319	0.0118	0.0533	0.1394	-2.8428
448.6147	0.0203	0.0533	2.6348	1.3977

449.0076	0.0267	0.0408	4.8809	2.2872
449.2162	0.0391	0.0704	0.2643	-1.9198
449.2392	0.0304	0.1066	0.2041	-2.2929
450.2422	0.0378	0.0533	0.1873	-2.4164
453.21	0.0302	0.0704	0.3988	-1.3265
454.1553	0.0226	0.0533	5.1777	2.3723
456.171	0.0150	0.0533	4.7394	2.2447
456.9415	0.0001	0.0304	15.6280	3.9661
457.2438	0.0223	0.1645	0.1508	-2.7295
460.1321	0.0124	0.0304	4.2015	2.0709
460.2046	0.0350	0.0533	0.1502	-2.7351
460.9538	0.0014	0.0304	8.3940	3.0694
461.2151	0.0268	0.0533	0.2531	-1.9822
462.1038	0.0455	0.0928	0.1327	-2.9143
462.8959	0.0361	0.0704	2.7211	1.4442
462.9383	0.0265	0.0533	2.9228	1.5473
463.9775	0.0154	0.0533	2.5756	1.3649
464.9618	0.0425	0.0704	2.9088	1.5404
464.9807	0.0412	0.0704	3.1347	1.6483
465.2581	0.0091	0.0408	0.2386	-2.0674
465.2705	0.0296	0.1066	0.0956	-3.3866
466.0042	0.0045	0.0304	4.6060	2.2035
466.1158	0.0272	0.0533	0.4680	-1.0953
466.902	0.0031	0.0453	0.1200	-3.0593
466.9898	0.0017	0.0304	14.4610	3.8541
467.2506	0.0127	0.0704	0.1903	-2.3937
470.0245	0.0019	0.0304	5.7242	2.5171
470.7286	0.0350	0.1066	2.3071	1.2061
471.0704	0.0050	0.0304	3.5776	1.8390
475.2562	0.0089	0.0533	0.1281	-2.9652
476.2578	0.0452	0.1066	0.4060	-1.3004
477.0083	0.0177	0.0704	0.0867	-3.5271
478.9304	0.0352	0.0704	3.8224	1.9345
480.5623	0.0112	0.0568	0.0957	-3.3846
480.6166	0.0346	0.0704	2.9106	1.5413
481.9791	0.0127	0.0704	5.9188	2.5653
482.9631	0.0002	0.0304	35.9610	5.1684
483.9683	0.0001	0.0304	5.4573	2.4482
483.9809	0.0004	0.0304	5.4698	2.4515
485.9645	0.0007	0.0304	8.6325	3.1098
485.9787	0.0020	0.0304	8.6344	3.1101
487.9787	0.0076	0.0304	6.9857	2.8044
488.9817	0.0006	0.0304	3.8690	1.9520

490.848	0.0484	0.1066	3.9642	1.9870
491.2268	0.0121	0.0568	0.2687	-1.8960
491.2868	0.0346	0.1066	0.1318	-2.9233
493.0531	0.0146	0.0304	3.2221	1.6880
493.2654	0.0201	0.1066	0.2015	-2.3112
494.0369	0.0337	0.0704	12.9960	3.7000
500.609	0.0033	0.0453	0.2003	-2.3195
502.5525	0.0174	0.0408	2.2549	1.1731
503.2275	0.0468	0.1066	0.2488	-2.0067
503.2581	0.0383	0.0533	2.2467	1.1678
503.9725	0.0008	0.0304	11.6200	3.5386
505.9621	0.0015	0.0304	4.8864	2.2888
505.9733	0.0003	0.0304	18.3710	4.1994
505.9887	0.0004	0.0304	5.3502	2.4196
506.0034	0.0007	0.0304	10.1520	3.3437
506.573	0.0431	0.1066	2.0321	1.0229
506.9921	0.0002	0.0304	17.2370	4.1074
507.9596	0.0193	0.0533	3.2528	1.7017
507.9934	0.0004	0.0304	10.8690	3.4421
509.0256	0.0457	0.0704	2.8767	1.5244
509.2963	0.0421	0.1066	0.1364	-2.8737
511.2746	0.0189	0.1066	0.2119	-2.2389
512.0666	0.0492	0.0704	2.4644	1.3012
513.2286	0.0458	0.0738	0.2425	-2.0438
515.9177	0.0138	0.0408	5.6128	2.4887
516.9537	0.0259	0.1066	0.2701	-1.8882
517.9146	0.0349	0.1066	6.9318	2.7932
519.1323	0.0414	0.0704	3.6190	1.8556
519.2808	0.0042	0.0304	0.0752	-3.7323
520.2849	0.0274	0.0408	0.2355	-2.0865
521.1479	0.0064	0.0408	5.5111	2.4623
521.9369	0.0211	0.0533	5.5189	2.4644
521.9835	0.0015	0.0304	22.4570	4.4891
523.1038	0.0376	0.1066	3.1059	1.6350
523.1653	0.0017	0.0304	5.7844	2.5322
524.0905	0.0381	0.1066	2.2340	1.1597
524.7183	0.0477	0.0704	3.2468	1.6990
525.3448	0.0350	0.0704	11.2180	3.4877
527.3072	0.0129	0.0533	0.2119	-2.2388
527.9587	0.0089	0.0304	3.2860	1.7163
527.9689	0.0015	0.0304	9.7623	3.2872
528.9732	0.0015	0.0304	5.3964	2.4320
530.0142	0.0107	0.0304	6.2709	2.6487

530.5863	0.0245	0.0533	2.9386	1.5551
530.9685	0.0392	0.0533	4.0320	2.0115
532.7577	0.0018	0.0304	3.5367	1.8224
535.3142	0.0435	0.1066	0.1621	-2.6247
536.3205	0.0150	0.0408	0.2413	-2.0508
537.2928	0.0140	0.0704	0.1973	-2.3417
538.3211	0.0307	0.0704	8.0689	3.0124
540.1408	0.0078	0.0304	3.5913	1.8445
541.124	0.0004	0.0304	9.6151	3.2653
541.1437	0.0077	0.0304	4.2114	2.0743
541.3384	0.0163	0.0304	4.8345	2.2734
542.1286	0.0005	0.0304	8.6135	3.1066
543.9334	0.0186	0.0533	3.0914	1.6282
543.9463	0.0005	0.0304	14.1780	3.8256
545.2967	0.0136	0.0704	0.1817	-2.4601
545.9683	0.0016	0.0304	7.9870	2.9976
549.2484	0.0345	0.0704	0.2221	-2.1709
549.6853	0.0436	0.1066	2.1497	1.1042
549.9522	0.0040	0.0304	5.0969	2.3496
553.3236	0.0123	0.0704	0.1287	-2.9585
555.3011	0.0187	0.0704	0.2096	-2.2543
561.3575	0.0110	0.0304	0.4205	-1.2498
563.3088	0.0071	0.0533	0.1740	-2.5227
563.9338	0.0002	0.0304	24.0770	4.5896
564.3109	0.0344	0.0533	0.3535	-1.5001
565.0464	0.0364	0.0304	2.1251	1.0875
565.1407	0.0006	0.0304	9.1137	3.1880
565.9256	0.0059	0.0408	7.8915	2.9803
566.0515	0.0265	0.0704	3.8905	1.9600
567.1563	0.0018	0.0304	9.9902	3.3205
570.8903	0.0289	0.0704	4.6588	2.2200
571.0413	0.0275	0.1066	2.3353	1.2236
573.0871	0.0477	0.0738	0.4120	-1.2794
576.0319	0.0128	0.0304	2.1152	1.0808
576.1176	0.0294	0.0304	4.1614	2.0571
577.1044	0.0005	0.0304	19.7720	4.3054
577.1189	0.0418	0.1645	3.3498	1.7441
578.1058	0.0049	0.0304	17.2780	4.1109
578.1125	0.0317	0.0704	3.4613	1.7913
579.0262	0.0203	0.1066	2.4884	1.3152
579.0982	0.0002	0.0304	31.3770	4.9716
579.105	0.0326	0.0704	2.2038	1.1400
579.2795	0.0023	0.0304	0.3567	-1.4871

580.1031	0.0336	0.0704	5.3856	2.4291
581.3214	0.0100	0.0533	0.1792	-2.4806
585.916	0.0245	0.0704	7.3401	2.8758
585.9299	0.0148	0.0408	2.8647	1.5184
586.6049	0.0261	0.0533	0.4245	-1.2363
586.8395	0.0101	0.0304	2.5396	1.3446
587.1033	0.0118	0.0704	3.6963	1.8861
587.9144	0.0269	0.0408	2.1220	1.0854
592.1125	0.0239	0.0533	4.4496	2.1537
593.1159	0.0209	0.0408	2.7246	1.4461
595.0389	0.0127	0.0304	5.2157	2.3829
599.3313	0.0022	0.0304	0.1428	-2.8082
601.8915	0.0098	0.0408	4.3702	2.1277
607.3345	0.0049	0.0408	0.1500	-2.7374
607.9108	0.0221	0.0704	2.1268	1.0887
611.1351	0.0070	0.0304	0.2150	-2.2176
611.1476	0.0007	0.0304	8.6998	3.1210
612.1449	0.0023	0.0408	2.1895	1.1306
612.5074	0.0402	0.0704	2.7767	1.4734
612.5981	0.0191	0.0704	0.1896	-2.3990
614.4787	0.0335	0.1066	0.3751	-1.4145
616.0444	0.0354	0.1066	2.5067	1.3258
623.3649	0.0348	0.0408	0.1885	-2.4076
624.3109	0.0132	0.1066	0.4708	-1.0867
625.3438	0.0189	0.0704	0.1839	-2.4433
630.6238	0.0302	0.0704	2.1757	1.1215
631.046	0.0149	0.0408	3.6648	1.8737
631.5089	0.0053	0.0304	4.5976	2.2009
633.1278	0.0407	0.0704	3.9494	1.9816
633.3486	0.0310	0.0704	0.2351	-2.0886
634.3305	0.0170	0.0704	0.4674	-1.0973
635.0581	0.0058	0.0304	9.2396	3.2078
641.3744	0.0353	0.1645	0.2106	-2.2477
642.8015	0.0355	0.0738	0.3646	-1.4557
642.8903	0.0304	0.0533	0.3156	-1.6639
643.3555	0.0301	0.1066	0.2139	-2.2250
644.359	0.0440	0.1066	0.3719	-1.4268
650.0657	0.0388	0.0704	0.4472	-1.1611
651.3579	0.0160	0.0533	0.2352	-2.0882
652.3623	0.0087	0.0304	0.2868	-1.8019
656.0854	0.0168	0.0533	3.7112	1.8919
662.3236	0.0359	0.0304	0.3723	-1.4254
662.9693	0.0295	0.0704	2.9299	1.5509

672.0794	0.0019	0.0304	7.3256	2.8730
674.3954	0.0003	0.0304	3.6408	1.8643
678.0647	0.0497	0.1645	2.0893	1.0630
679.3313	0.0422	0.0533	0.2499	-2.0009
685.1773	0.0198	0.0533	2.2757	1.1863
695.3732	0.0256	0.1066	0.4134	-1.2743
696.3682	0.0325	0.0704	2.3337	1.2226
713.3954	0.0364	0.1066	0.2755	-1.8599
718.425	0.0053	0.0304	3.7411	1.9035
722.3522	0.0260	0.0533	2.8003	1.4856
726.3756	0.0150	0.0408	2.5645	1.3587
753.2385	0.0077	0.0533	2.4115	1.2699
757.4218	0.0189	0.0533	0.2819	-1.8267
759.4728	0.0453	0.0533	3.2164	1.6855
775.4327	0.0304	0.1066	0.2889	-1.7916
782.7977	0.0146	0.1066	2.2857	1.1926
783.4339	0.0235	0.1066	0.2838	-1.8171
799.4698	0.0339	0.0408	0.4055	-1.3022
801.451	0.0308	0.1645	0.2753	-1.8612
806.1799	0.0348	0.0704	3.4268	1.7768
808.1726	0.0250	0.0704	3.5916	1.8446
811.4068	0.0448	0.0304	0.3536	-1.4997
827.1367	0.0066	0.0304	5.6349	2.4944
828.1416	0.0038	0.0304	5.3500	2.4195
829.1375	0.0006	0.0304	4.4983	2.1694
832.1582	0.0026	0.0304	2.7332	1.4506
845.4757	0.0186	0.0704	0.2764	-1.8554
850.1671	0.0017	0.0304	9.1194	3.1889
851.1704	0.0027	0.0304	6.8434	2.7747
852.164	0.0218	0.0533	3.5773	1.8389
889.5021	0.0050	0.0304	0.2907	-1.7825
893.5823	0.0300	0.0704	2.6433	1.4023
917.5753	0.0301	0.0533	4.1765	2.0623
926.8146	0.0378	0.0704	2.0944	1.0665
930.1313	0.0095	0.0304	6.6259	2.7281
933.0158	0.0127	0.0304	3.8436	1.9425
936.6215	0.0175	0.0304	3.8205	1.9338
960.6242	0.0243	0.0533	5.4313	2.4413
977.5543	0.0409	0.1645	0.4365	-1.1959
1016.1707	0.0319	0.0533	3.6912	1.8841
1019.1609	0.0450	0.0704	2.6524	1.4073

Table S2. Hundred-sixty functionally annotated metabolites altered between WT and *Hyp* cortical bone with raw p-value, adjusted p-value, FC, log2(FC), and the location where metabolites visualize (osteoid and/or mineralized bone). The analysis is based on mean values (averages) of individuals (n = 5 per group).

compound name	raw p-value	adjusted p-value (Benjamini Hochberg)	FC	log2(FC)	localized in mineralized bone	localized in osteoid
Sulfate	0.0649	0.0704	2.1937	1.1334	+	+
Pyridine	0.1721	0.2384	0.3475	-1.5251	+	
Taurine	0.0437	0.1066	2.0239	1.0171		+
Thymine	0.1226	0.4363	0.3445	-1.5373		+
Aspartate	0.0559	0.0533	3.3228	1.7324		+
Malate	0.0456	0.0533	2.9482	1.5598		+
Oxobutanoate, Acetoacetate, Succinate semialdehyde	0.6457	0.7024	0.3751	-1.4148	+	+
Hydroxypyruvate, Acetyl phosphate	0.0340	0.0704	2.1965	1.1352		+
Glutamine	0.0639	0.0704	4.0842	2.0301		+
Indolequinone	0.3131	0.4363	2.3697	1.2447	+	+
Glutamate, Hydroxyglutamate semialdehyde	0.0541	0.0704	2.7990	1.4849		+
Sulfinylpyruvate	0.7018	0.8485	0.1748	-2.5166	+	+
Fumarate	0.0140	0.0533	2.1681	1.1165		+
Dehydrodeoxy-fuconate	0.0429	0.1066	2.0422	1.0302	+	+
Homocysteic acid	0.1428	0.2384	2.3171	1.2123	+	+
Guanidinobutanoate	0.0152	0.0533	0.1663	-2.5881	+	+
Phosphoenolpyruvate	0.0074	0.0304	5.2281	2.3863		+
Beta-Carboline	0.1111	0.3271	0.3175	-1.6550	+	
Glycerone phosphate, Glyceraldehyde phosphate	0.0026	0.0304	9.0750	3.1819		+
sn-Glycerol phosphate	0.0212	0.0533	2.2209	1.1512		+
Homocysteine	0.3485	0.2671	0.2095	-2.2549	+	+
Phenol sulphate	0.0109	0.0304	5.7887	2.5333	+	+
N-Acetylornithine	0.7755	0.8485	43.3048	5.4365	+	+
Hydroxyanthranilate	0.1171	0.0704	3.0097	1.5896		+

N-Acetylaspartate	0.1052	0.1066	2.7072	1.4368		+
Glucuronolactone	0.0567	0.0704	2.7285	1.4481		+
Pyrophosphate	0.0128	0.0304	2.0976	1.0687		+
4-Hydroxyphenyl)pyruvate, Hydroxy-(4-hydroxyphenyl)propenoate	0.1144	0.1645	2.4916	1.3171	+	+
Methionine	0.0134	0.0533	6.1722	2.6258		+
Phosphoglycerate	0.0015	0.0304	19.4458	4.2814		+
O-methoxycatechol-O-sulphate	0.3102	0.7024	7.3976	2.8871	+	+
Xanthine	0.0277	0.0533	8.1834	3.0327	+	+
Diethyl glutarate, Diethyl methylsuccinate	0.7970	1.0000	0.3991	-1.3253	+	
Kynurenic acid	0.3004	0.7024	64.7473	6.0167	+	+
chondroitin sulfate E proteoglycan	0.4723	0.7024	4.7508	2.2482		+
N-Acetylhistamine	0.2785	0.3507	0.4151	-1.2686	+	+
Citrate,Isocitrate	0.0170	0.0304	3.0837	1.6247		+
Arginine	0.2501	0.4363	0.2475	-2.0144	+	
N-Acetylhistidine	0.0591	0.1066	0.3892	-1.3615	+	
beta-Alanyllysine	0.1615	0.1645	0.3261	-1.6167	+	
Cysteate	0.0350	0.0533	4.6982	2.2321	+	+
Hydroxykynurenine	0.0036	0.0304	0.1432	-2.8041	+	+
Pro-Asn	0.0568	0.1066	0.3212	-1.6384	+	+
Cys-Gly	0.0043	0.0304	7.1650	2.8410		+
Deoxyribose phosphate	0.0042	0.0304	2.8907	1.5314	+	+
Fructose, Glucose, Mannose, myo-Inositol, Galactose	0.0002	0.0304	2.8282	1.4999		+
Bisnorbiotin	0.6810	1.0000	0.2510	-1.9941	+	+
Formylkynurenine	0.1416	0.3271	0.1082	-3.2077	+	
O-Phosphoserine	0.0690	0.1066	3.7089	1.8910		+
Phosphomevalonate	0.3058	0.2384	0.3909	-1.3551	+	
Pyroglutamylvaline	0.3718	0.3271	0.3753	-1.4140		+
Ribulose phosphate, Xylulose phosphate	0.0136	0.0304	2.2434	1.1657		+
Phosphoarginine	0.8759	0.8485	0.4372	-1.1936		+

PPPi	0.0570	0.0704	2.2047	1.1406		+
Cytidine	0.3023	0.1645	0.3471	-1.5268		+
Gln-Pro	0.1837	0.2384	0.2814	-1.8293		+
Cys-Gln	0.1363	0.2384	2.4355	1.2842		+
2-Oxo-3-hydroxy-4-phosphobutanoate	0.0158	0.0408	4.2230	2.0783		+
Deoxyadenosine	0.2830	0.4363	0.2806	-1.8334		+
Val-His	0.1168	0.1066	0.2925	-1.7733	+	+
Palmitoleic acid	0.1048	0.2384	0.0243	-5.3644	+	
Hydroxytryptophan	0.1371	0.1066	0.4555	-1.1345	+	+
His-Thr	0.1295	0.1066	0.4218	-1.2453	+	+
(Iso)Palmitic acid	0.7219	0.5641	0.3598	-1.4749	+	+
Methylcytidine	0.3551	0.3271	0.4042	-1.3068		+
4a-Hydroxytetrahydrobiopterin	0.1609	0.1645	0.2251	-2.1515		+
(Iso)Leu-Gln	0.3777	0.4363	0.3438	-1.5403		+
Asn-Gln	0.1863	0.3271	0.2968	-1.7524		+
Gln-Asn	0.1026	0.1645	4.2836	2.0988		+
Gly-Try	0.3252	0.4363	0.3516	-1.5082		+
Asn-Met	0.0109	0.0408	2.1598	1.1109		+
Deoxyuridine	0.5851	1.0000	0.2402	-2.0576		+
Phosphoglyceroyl phosphate, Bisphosphoglycerate	0.0053	0.0533	6.4691	2.6936		+
Ribose phosphate	0.0000	0.0304	11.9448	3.5783		+
Acetylcarnosine	0.1062	0.1066	0.3015	-1.7297	+	+
Ophthalmate	0.2297	0.2384	0.3852	-1.3763		+
Arn-Pro	0.1166	0.2384	0.2452	-2.0280		+
Arn-Val	0.1170	0.2384	0.2857	-1.8074		+
Gln-Gln	0.3341	0.3271	0.4105	-1.2845		+
Phosphogluconate	0.1328	0.1066	3.8374	1.9401		+
Asn-Phe	0.3655	0.3271	0.2663	-1.9089		+
Linoleate	0.8566	0.7024	0.2529	-1.9832		+
Oleic acid	0.8425	0.5641	0.2209	-2.1784		+

His-Gln	0.1112	0.3271	0.2110	-2.2444		+
Lys-His	0.0824	0.1645	0.1489	-2.7478		+
Tyr-Cys	0.6007	1.0000	0.4553	-1.1351	+	+
Stearic acid	0.8888	0.7024	0.4644	-1.1066	+	+
Arn-Hydroxyproline	0.1337	0.1645	0.2140	-2.2244		+
Arn-(Iso)Leu	0.3984	0.4363	0.3344	-1.5803		+
Sedoheptulose phosphate	0.0007	0.0304	9.5734	3.2590		+
Galactosylglycerol	0.4999	0.8485	0.2677	-1.9014		+
Try-Ser	0.0435	0.0533	0.2689	-1.8950	+	+
Phe-GGlu, Phe-Gln	0.5207	0.4363	0.4203	-1.2504		+
Phosphonogluconolactone	0.0359	0.0704	2.1446	1.1007	+	+
Methylguanosine	0.0453	0.1590	0.2140	-2.2246	+	+
N-Acetylserotonin sulfate	0.4483	0.7024	5.3317	2.4146	+	
LysoPA(8:0/0:0)	0.1727	0.2384	0.1014	-3.3016	+	
Glutathione	0.0009	0.0304	2.5062	1.3255		+
Ribose bisphosphate, Glucuronic acid phosphate	0.0592	0.1066	3.2939	1.7198		+
Xanthosine	0.3050	0.5641	2.3683	1.2438		+
Arn-Phe	0.1458	0.2384	0.2463	-2.0216		+
Inosine 2',3'-cyclic phosphate	0.0427	0.0704	2.9250	1.5485		+
dAMP	0.4868	0.4363	0.3598	-1.4747		+
Gln-Trp,Trp-Gamma-glu	0.3312	0.3271	0.3263	-1.6159		+
Lys-Trp	0.5618	0.8485	0.3904	-1.3570		+
Arn-Tyr	0.2425	0.2384	0.3299	-1.5999		+
S-Sulfanylglutathione	0.0123	0.0408	7.3140	2.8707		+
myo-Inositol bisphosphate, Tagatose bisphosphate, Glucose bisphosphate, myo-Inositol bisphosphate, Fructose bisphosphate	0.0071	0.0304	3.4229	1.7752		+
Leukotriene A4	0.4425	0.7024	37.7399	5.2380	+	+
Xanthylic acid	0.0244	0.0533	5.6869	2.5076	+	+
IMP	0.0154	0.0408	3.4167	1.7726		+

MG(0:0/i-16:0/0:0)	0.7397	1.0000	0.3590	-1.4778		+
Phosphoribosyl pyrophosphate	0.0006	0.0304	11.0399	3.4647		+
Dolichyl diphosphate	0.5349	0.8485	0.3702	-1.4335	+	
S-Adenosylmethionine	0.5265	0.8485	2.2827	1.1907	+	
Prostaglandin G2	0.6984	0.7024	0.4155	-1.2671		+
LysoPA(P-16:0e/0:0)	0.2942	0.5641	0.1382	-2.8548		+
hexadecyl-glycero-phosphate	0.1077	0.1645	0.1252	-2.9972		+
dTDP	0.3408	0.3271	0.4262	-1.2303	+	+
CDP	0.0304	0.0533	2.8480	1.5099		+
1-(9Z-hexadecenoyl)-glycero-3-phosphate, 1-palmitoyl-dihydroxyacetone-phosphate	0.2596	0.2384	0.4214	-1.2466	+	+
dADP	0.1857	0.2384	0.2590	-1.9492	+	+
DHAP(18:0e)	0.6358	0.3271	0.3118	-1.6811		+
Adenylyl sulfate	0.0022	0.0304	9.2628	3.2114		+
ADP, Adenosine 3',5'-bisphosphate, dGDP	0.0011	0.0304	2.7263	1.4469		+
IDP	0.0092	0.0408	16.2787	4.0249		+
CMP-2-aminoethylphosphonate	0.1027	0.1066	2.6364	1.3986		+
LysoPA(0:0/18:2(9Z,12Z))	0.4572	0.4363	0.3866	-1.3710		+
Dolichyl b-D-glucosyl phosphate	0.4227	0.4363	0.4027	-1.3123	+	+
Heptadecanoylglycerophosphoethanolamine, LysoPC(14:0/0:0)	0.3004	0.4363	0.1907	-2.3909	+	
1-Phosphatidyl-1D-myo-inositol 3-phosphate	0.6084	0.3271	2.7091	1.4378	+	+
N-Docosahexaenoyl phenylalanine	0.2063	0.4363	0.2026	-2.3033	+	+
PA(8:0/12:0)	0.1293	0.1645	0.3649	-1.4543	+	+
CTP	0.0127	0.0704	5.0702	2.3420		+
UTP	0.0002	0.0304	30.9234	4.9506		+
ATP, dGTP	0.0004	0.0304	4.8908	2.2901		+
GTP	0.0079	0.0304	18.8604	4.2372		+
LysoPE(0:0/20:1(11Z))	0.4062	0.5641	0.3943	-1.3427		+
LysoPA(24:1(15Z)/0:0)	0.0015	0.0304	18.8605	4.2373		+
PA(10:0/i-14:0), PA(8:0/16:0), PA(i-12:0/i-12:0)	0.2097	0.1645	0.3685	-1.4403	+	+

UDP-glucose, UDP-alpha-D-galactose	0.0364	0.0304	2.0188	1.0135		+
1-hexadecanoyl-sn-glycero-3-phospho-D-myo-inositol, 1-Palmitoylglycerophosphoinositol	0.2316	0.2384	0.3555	-1.4922	+	
UDP-glucuronate	0.0203	0.1066	2.6720	1.4179		+
DG[C35H68O5]	0.7714	0.4363	2.2656	1.1799	+	+
CerP(d18:1/16:0)	0.2385	0.4363	0.2802	-1.8357	+	
Glutathione disulfide	0.0407	0.0704	3.4379	1.7815		+
PA[C35H69O8P]	0.4177	0.5641	0.2164	-2.2083	+	
PS(14:0/14:0)	0.3934	0.4363	0.4186	-1.2564	+	
PG(i-12:0/18:2(9Z,11Z))	0.3197	0.4363	0.4440	-1.1715	+	+
PA[C39H75O8P]	0.9226	0.8485	0.4026	-1.3127		+
ADP-Ribosyl-L-arginine	0.0503	0.0704	2.2105	1.1444	+	
PE or PC [C39H76NO8P]	0.5249	0.8485	0.3787	-1.4008	+	+
PE or PC [C39H78NO8P]	0.7192	0.5641	0.3506	-1.5120	+	+
PA[C41H75O8P]	0.5381	0.7024	0.3369	-1.5695		+
PE or PC [C41H80NO8P]	0.3842	0.7024	0.3292	-1.6029		+
PE or PC [C43H82NO8P]	0.9850	1.0000	0.4124	-1.2779	+	+
SM(d18:1/23:0)	0.3303	0.7024	0.2411	-2.0523	+	+
TG[C51H86O6]	0.5662	1.0000	0.2021	-2.3072	+	
3-Methylbutanoyl-CoA, (S)-2-Methylbutanoyl-CoA	0.0017	0.0304	8.5468	3.0954		+
TG[C55H98O6]	0.1139	0.1590	0.1290	-2.9544	+	+
